# Modulating metabolic signatures to mitigate cabozantinib resistance in *FLT3*-ITD acute myeloid leukemia cell models

**DOI:** 10.1038/s41420-026-02957-8

**Published:** 2026-02-17

**Authors:** Yu-Hsuan Fu, Kit Man Ng, Chi-Yang Tseng, Ang-Chu Huang, Chin-Hsien Tu, Wen-Chun Chen, Pei-Chi Lang, Hsiung-Fei Chien, Liang-In Lin

**Affiliations:** 1 Department of Clinical Laboratory Sciences and Medical Biotechnology, National Taiwan University, Taipei, Taiwan; 2 Department of Surgery, Taipei Medical University Hospital, Taipei, Taiwan; 3 TMU Center for Cell Therapy and Regeneration Medicine, Taipei Medical University, Taipei, Taiwan; 4 Department of Laboratory Medicine, National Taiwan University Hospital, Taipei, Taiwan

**Keywords:** Cancer metabolism, Acute myeloid leukaemia

## Abstract

Drug resistance remains a major challenge in treating acute myeloid leukemia (AML), despite advancements in targeted therapies. We established cabozantinib-resistant FLT3-ITD^+^ AML cell lines (MV4-11-XR, Molm13-XR) from parental MV4-11 and Molm13 cells. In addition to resistance to cabozantinib, they also exhibited resistance to FDA-approved sorafenib and quizartinib with substantial increases in IC_50_. The FLT3 D835Y mutation emerged in both cell lines, while an additional 1.3 kb deletion in *FLT3 (FLT3*¹^.^³) was present in MV4-11-XR cells. Both resistant cells displayed higher proliferation rates and increased colony formation, as well as increased phosphorylation of FLT3 and its downstream signaling molecules, including ERK, STAT5, and AKT. Transcriptomic analysis identified 1113 and 1057 differentially expressed genes (DEGs) in MV4-11-XR and Molm13-XR, respectively, compared with their parentals, of which 81 and 74 DEGs are metabolic-related. Further metabolic assays confirmed that cabozantinib resistance was associated with significant metabolic alterations, including enhanced glycolysis with increased glucose uptake, lactate production, GAPDH activity, and glycolytic gene expression, as well as impaired oxidative phosphorylation and reduced mitochondria mass. Further in silico drug screening and in vitro experiments demonstrated that PI3K/mTOR dual inhibitor omipalisib and HSP90 inhibitor radicicol effectively reversed the metabolic reprogramming in cabozantinib-resistant cells. Moreover, both omipalisib and radicicol exhibited synergistic effects with cabozantinib, highlighting their therapeutic potential. Overall, we identified metabolic dysregulation as a hallmark of cabozantinib resistance and suggested that targeting metabolic vulnerabilities with PI3K/mTOR or HSP90 inhibitors could be an option to mitigate drug resistance.

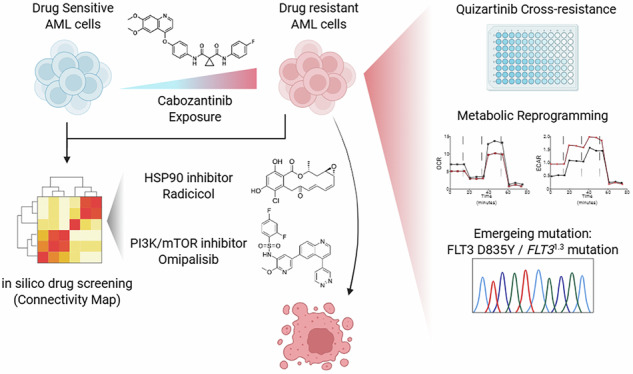

## Introduction

Acute myeloid leukemia (AML) is a hematological malignancy characterized by genetic heterogeneity, leading to uncontrolled proliferation and impaired myeloid differentiation. While targeted therapies against *FLT3*, *IDH1*, and *IDH2* mutations have improved survival in certain patient subsets, the emergence of acquired drug resistance remains a major challenge [1, 2]. Therefore, identifying the molecular features associated with resistance and developing alternative therapeutic strategies to exploit these vulnerabilities are critical for improving AML treatment outcomes.

Cabozantinib, an oral multi-tyrosine kinase inhibitor (TKI) targeting MET, VEGFR2, RET, KIT, TIE-2, and FLT3, has been extensively studied in solid tumors [3]. The FDA has approved cabozantinib for the treatment of advanced/progressive metastatic medullary thyroid cancer [4], advanced renal cell carcinoma [5, 6], and advanced and progressive hepatocellular carcinoma [7]. We previously demonstrated, for the first time, the in vitro and in vivo efficacy of cabozantinib against *FLT3*-ITD [8] and KIT-driven t(8;21) AML [9]. Additionally, we showed that cabozantinib promotes erythroid differentiation in K562 leukemia cells [10]. Moreover, a clinical trial suggested that cabozantinib is well tolerated in patients with *FLT3*-ITD AML [11], further emphasizing its potential in targeting specific AML subtypes. Although cabozantinib has demonstrated efficacy in overcoming resistance in cytarabine-resistant AML [12], radioiodine-resistant thyroid cancer [13], and castration-resistant prostate cancer [14], concerns remain regarding its potential to induce resistance following treatment. Understanding the mechanisms underlying acquired resistance and developing targeted strategies to counteract it are essential for optimizing long-term therapeutic efficacy.

In this study, we established two cabozantinib-resistant cell lines, Molm13-XR and MV4-11-XR, derived from *FLT3*-ITD-positive AML cell lines Molm13 and MV4-11, respectively, by exposing cells to increasing concentrations of cabozantinib. These resistant cells exhibited cross-resistance to other two FDA-approved TKIs, quizartinib and sorafenib. Subsequently, we characterized the genomic and transcriptomic alterations in Molm13-XR and MV4-11-XR cells. Specifically, we identified the emergence of the FLT3 tyrosine kinase domain mutation D835Y in both resistant cell lines, along with a unique *FLT3*¹^.^³ deletion in MV4-11-XR cells. Furthermore, we found that cabozantinib resistance was associated with metabolic reprogramming, including enhanced glycolysis and impaired oxidative phosphorylation. Using in silico drug screening via connectivity map (CMap) analysis, we demonstrated that the HSP90 inhibitor radicicol and the PI3K/mTOR dual inhibitor omipalisib could effectively reverse the glycolytic shift in cabozantinib-resistant cells. These findings provide critical insights into the consequences of TKI resistance and metabolic dysregulation while highlighting alternative therapeutic strategies to mitigate resistance in AML.

## Results

### Characterization of cabozantinib-resistant AML cells

**Fig. 1:**
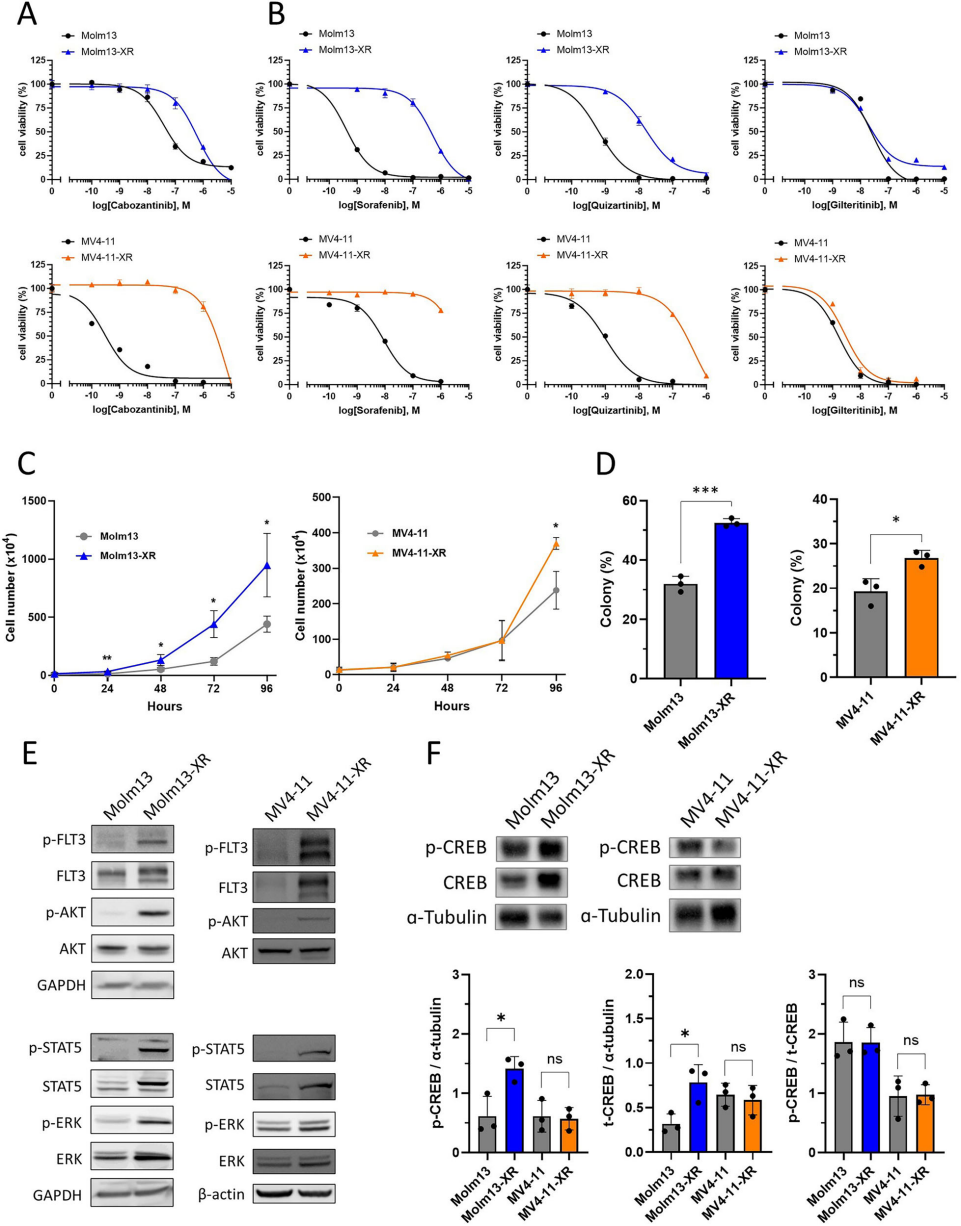
Characterization of cabozantinib-resistant AML cells. **A**, **B** Cell viability of indicated cell lines was evaluated by MTS assay after treatment with indicated TKIs for 72 h. **C** Assessment of the growth of indicated cell lines by PI (propidium iodide) exclusion method combined with flow cytometry. The doubling times of Molm13-XR and MV4-11-XR cells were 16.99 h and 18.66 h, respectively, which were shorter than 20.84 h of Molm13 and 19.59 h of MV4-11 cells. **D** The colony formation rate of indicated cell lines after 14 days of culture. **E**, **F** The indicated phosphorylation and protein levels in Molm13, Molm13-XR, MV4-11, and MV4-11-XR were analyzed by immunoblotting. GAPDH, β-actin, or α-tubulin was used as the loading control. Representative immunoblots of three independent experiments with similar results are shown. Bar charts showing the ratios between levels of p-CREB, t-CREB, and α-tubulin in the indicated cells. All experiments were performed in at least two independent experiments each performed in triplicate. *p < 0.05, **p < 0.01, ***p < 0.001 compared with parental cells.

The half maximal inhibitory concentrations (IC_50_) of cabozantinib were 602.0 nM and 6435 nM for Molm13-XR and MV4-11-XR, respectively, compared to 39.3 nM and 0.3 nM for their parental cells (Fig. 1A). Cytotoxicity assays further confirmed that Molm13-XR and MV4-11-XR exhibited cross-resistance to sorafenib and quizartinib, which are classified as type II TKI, but remained sensitive gilteritinib, which is classified as type I TKI (Fig. 1B). In addition, both resistant cell lines displayed higher proliferation rates and increased colony formation abilities compared to their parental counterparts (Fig. 1C-D, Supplementary Figure S1A). Western blot analysis revealed increased phosphorylation of FLT3 and its downstream signaling molecules, including ERK, STAT5, and AKT, in Molm13-XR and MV4-11-XR cells compared to their parental cells (Fig. 1E). Further phosphoprotein array analysis and immunoblotting demonstrated that the levels of total CREB and phosphorylated CREB (Ser133) were increased in Molm13-XR cells, but not in MV4-11-XR cells, compared to their parental cells (Fig. 1F, Supplementary Figure S1B). However, there were no significant differences in the percentage of various cell surface markers between the resistant and parental cells (Supplementary Table S1).

Next, we identified the *FLT3* c.2503 G > T mutation (FLT3 D835Y) in both resistant cell lines (Supplementary Fig. S1C) with an increasing variant allele frequency (VAF) of the mutant allele from 3% to 35% in Molm13-XR and from 5% to 99% in MV4-11-XR cells, respectively (Supplementary Fig. S1D). In addition, a transcript harboring an exon 20 deletion was noted in MV4-11-XR; further fragment analysis revealed that the deletion frequency increased from 10.2% to 46.6% in MV4-11-XR cells (Supplementary Fig. S2A). No mutations were found in the consensus sequences at the exon-intron splice junctions (Supplementary Fig. S2B), ruling out alternative splicing. Further sequencing of genomic DNA spanning intron 19 to intron 20 confirmed the absence of a 1.3 kb fragment in MV4-11-XR cells, which resulted in the loss of exon 20, and is here designated as the *FLT3*^1.3^ mutation (Supplementary Fig. S2C). Sanger sequencing of cDNA from MV4-11 and MV4-11-XR cells demonstrated that the parental MV4-11 cells expressed wild-type D835 on both alleles, whereas MV4-11-XR cells harbored the D835Y mutation on one allele and the *FLT3*^1.3^ mutation on the other allele (Supplementary Fig. S2D–E), consistent with the genomic findings. These findings indicate the clonal selection of the D835Y and *FLT3*^1.3^ alleles during cabozantinib exposure.

To assess whether cabozantinib resistance is associated with the selected *FLT3* mutations, 32D and various 32D-derived cells, including 32D-ITD cells, 32D-ITD N676D cells, 32D-ITD F691L cells, 32D-ITD D835Y cells, and 32D-ITD Y842H cells, were used. These cell lines represented 32D cells transduced with *FLT3*-ITD alone or in combination with N676D, F691L, D835Y, or Y842H in 32D cells, respectively. The IC₅₀ value of parental 32D cells was 46.22 mM, while those in 32D-ITD cells, 32D-ITD N676D cells, 32D-ITD F691L cells, 32D-ITD D835Y cells, and 32D-ITD Y842H cells were 77.61 nM, 4.57 µM, 6.17 µM, 1.10 mM, and 0.30 mM, respectively (Supplementary Fig. S3A). Based on previous studies of cabozantinib administration in mice with a threshold of 10 µM [15], 32D-ITD cells, 32D-ITD N676D cells, and 32D-ITD F691L cells were identified as sensitive to cabozantinib, while 32D-ITD D835Y and 32D-ITD Y842H cells were cabozantinib-resistant. Consequently, these results suggest that the emerged FLT3 D835Y mutation found in resistant cells of parental cells might be associated with cabozantinib resistance.

### Transcriptomic analysis and functional assays reveal dysregulation of glycolysis and oxidative phosphorylation in cabozantinib-resistant cells

**Fig. 2:**
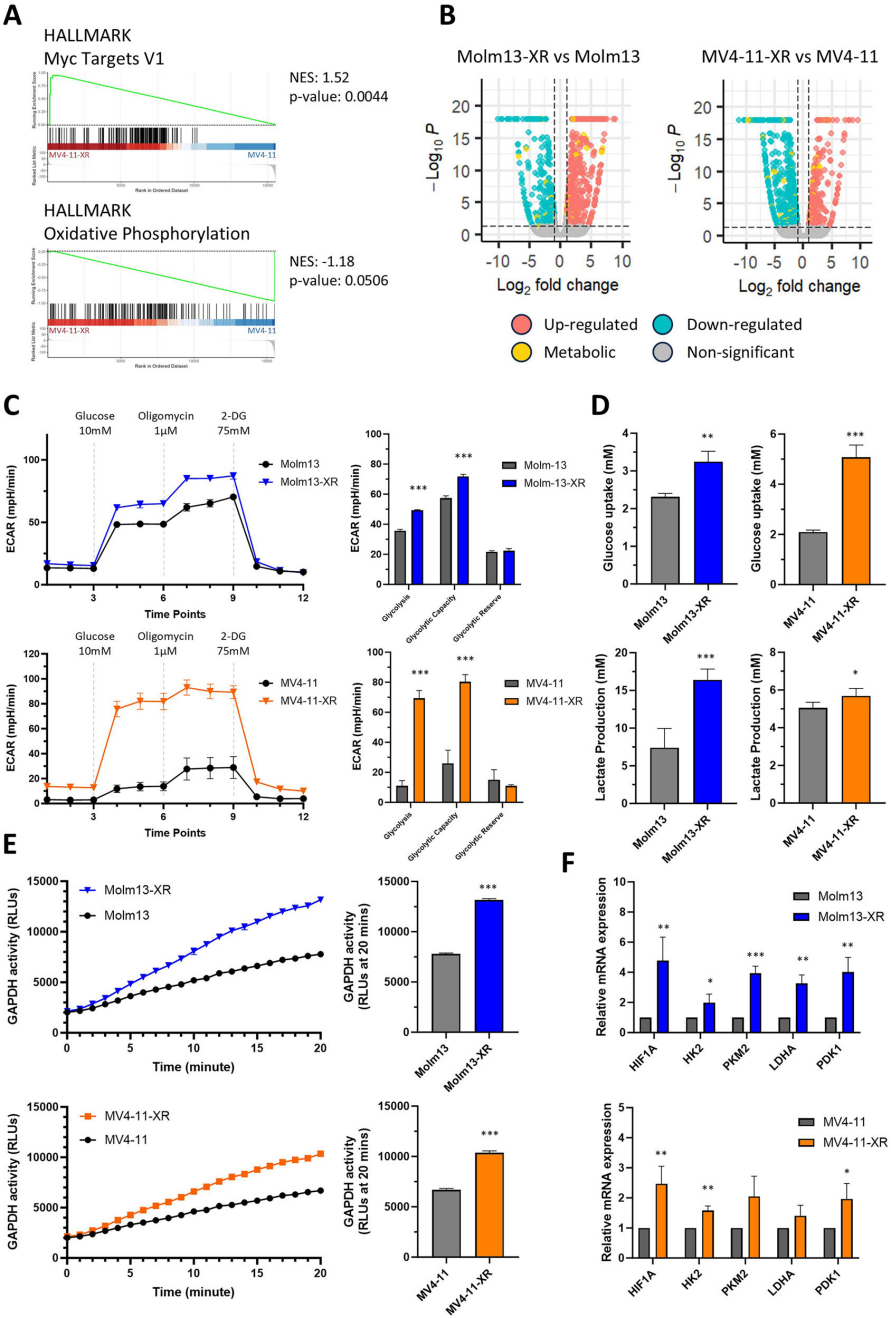
Analysis of transcriptome and glycolytic features of cabozantinib-resistant cells. **A** Enrichment plot from the gene-set enrichment analysis (GSEA). The normalized enrichment scores (NES) of indicated pathways are calculated based on log (Fold Change) *log(*p*-value) between MV4-11-XR and MV4-11. **B** Volcano plot showing DEGs that are upregulated (red dots) or downregulated (blue dots) of resistant cells compared to parental cells. DEGs related to the KEGG metabolic pathway were shown as yellow dots. **C** Seahorse Glycolysis Stress Test was performed to examine basal glycolysis, glycolytic capacity, and glycolytic reserve of indicated cells. **D**, **E** Glucose consumption, lactate production, and GAPDH activity of indicated cells. **F** RT-qPCR analysis of indicated genes in Molm13, Molm13-XR, MV4-11, and MV4-11-XR cells; 18 s rRNA was used for normalization. Data are representative of two independent experiments each performed in triplicate. *p < 0.05, **p < 0.01, ***p < 0.001 compared with parental cells.

To unravel the molecular signatures of cabozantinib resistance, we performed transcriptomic analysis using RNA sequencing (RNA-seq) on MV4-11-XR, Molm13-XR, and their parental cells. We identified 1113 differentially expressed genes (DEGs) in MV4-11-XR compared to its parental MV4-11 cells, including 366 upregulated and 725 downregulated genes, based on the criteria of *P* ≤ 0.05 and log_2_ fold change (FC) ≥ 1 or ≤ -1. Similarly, 1057 DEGs were identified between Molm13 and Molm13-XR cells, including 650 upregulated and 407 downregulated genes in the resistant cells. Gene set enrichment analysis (GSEA) revealed that the “Myc Targets V1” pathways was significantly enriched in resistant cells, whereas “oxidative phosphorylation” was enriched in the parental MV4-11 cells (Fig. 2A). KEGG analysis further showed that 81 of the 1113 DEGs in MV4-11-XR and 74 of the 1057 DEGs in Molm13-XR were linked to metabolic pathways (Supplementary Table S2), highlighting metabolic dysregulation as a hallmark of cabozantinib resistance (Fig. 2B).

To further elucidate the role of metabolic reprogramming in cabozantinib resistance, we assessed glycolysis, oxidative phosphorylation, and mitochondrial function. Glycolysis stress tests demonstrated significant increases in both basal glycolytic rate and glycolytic capacity in the resistant cell lines (Fig. 2C), accompanied by enhanced glucose uptake and lactate production (Fig. 2D) as well as elevated GAPDH activity (Fig. 2E). Furthermore, the expression levels of key glycolysis-related genes—including *HIF1A*, *HK2*, *PKM2*, *LDHA*, and *PDK1*—were markedly upregulated in the resistant cells compared to their parental counterparts (Fig. 2F).

**Fig. 3:**
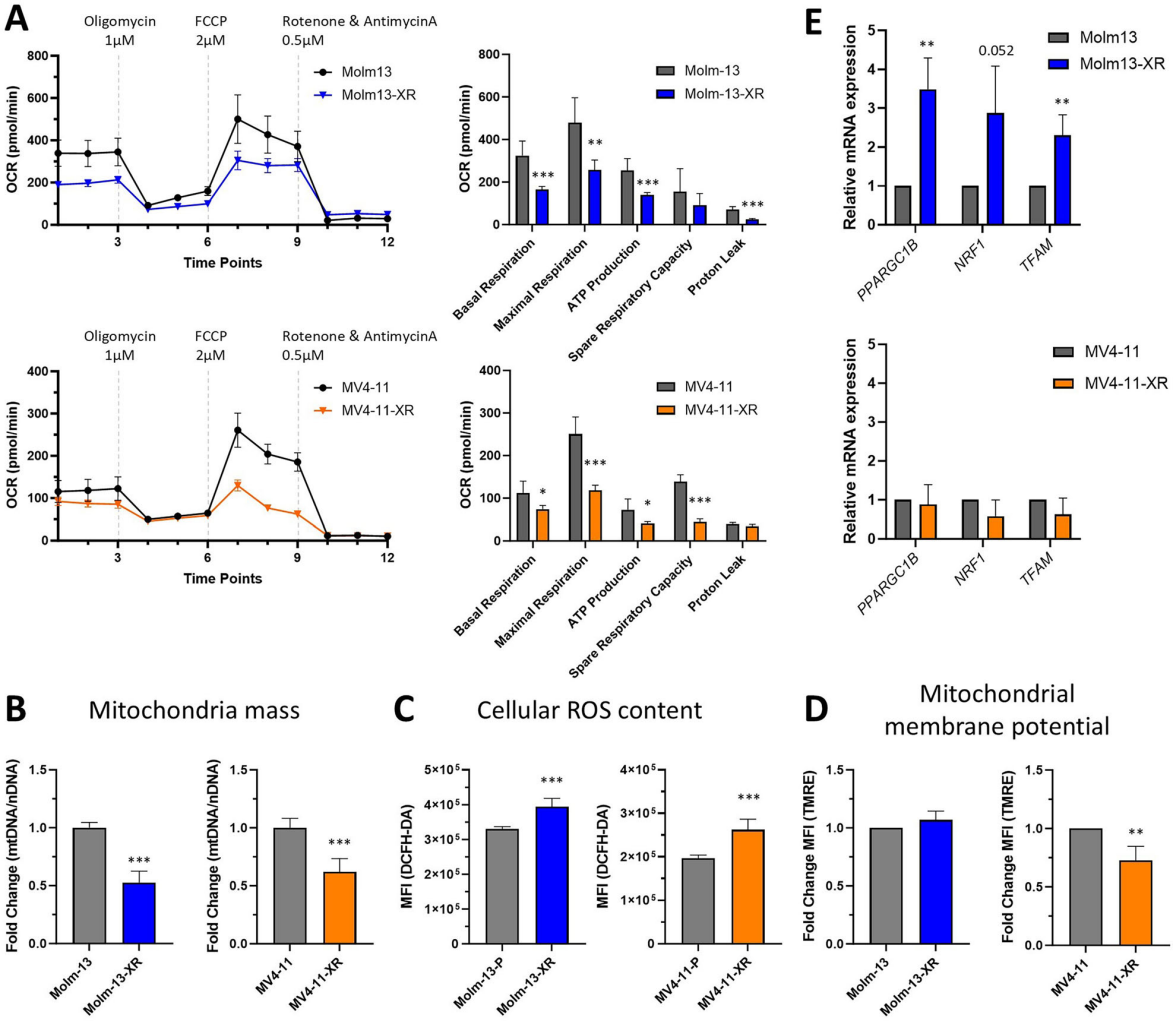
Evaluation of mitochondrial function in cabozantinib-resistant cells. **A** Seahorse Mito Stress Test was performed to measure basal respiratory, spare respiratory capacity, proton leak, and ATP-linked respiratory in indicated cells. **B** Mitochondrial mass of indicated cells was evaluated by q-PCR assay to compare DNA amount of *tRNA-Leu* (*UUR*) and *B2M*. **C** Intracellular reactive oxygen species (ROS) levels were measured by DCFH-DA staining coupled with flow cytometry. **D** Mitochondria membrane potentials were evaluated by TMRE staining coupled with flow cytometry. **E** RT-qPCR analyses of indicated genes in Molm13, Molm13-XR, MV4-11, and MV4-11-XR cells; 18 s rRNA was used for normalization. Data are representative of two independent experiments each performed in triplicate. *p < 0.05, **p < 0.01, ***p < 0.001, compared with parental cells.

Mito Stress tests, performed using the Seahorse analyzer, revealed impaired mitochondrial oxidative phosphorylation in both Molm13-XR and MV4-11-XR cells, as evidenced by reductions in both basal and maximal respiration compared to their parental counterparts (Fig. 3A). Regarding mitochondrial biogenesis, both resistant cell lines exhibited relatively less mitochondrial mass (Fig. 3B) but higher levels of intracellular reactive oxygen species (ROS) compared to their parental cells (Fig. 3C). Mitochondrial membrane potential was also significantly reduced in MV4-11-XR cells (Fig. 3D), whereas Molm13-XR cells displayed increased expression of genes associated with the PGC-1/ERR network of mitochondrial biogenesis, including *PPARGC1B*, *NRF1*, and *TFAM* (Fig. 3E). Furthermore, to elucidate the impact of emergent FLT3 secondary mutations on metabolic reprogramming, we assessed metabolic alterations in 32D-ITD cells alone or in combination with FLT3 D835Y. We found that the acquisition of the FLT3 D835Y mutation not only impaired mitochondrial oxidative phosphorylation (Supplementary Fig. S3B) but also reduced glycolytic activity (Supplementary Fig. S3C).

Taken together, these results indicate that metabolic dysregulation, specifically, hyperactivated glycolytic metabolism accompanied by impaired mitochondrial oxidative phosphorylation is a hallmark of cabozantinib resistance.

### Connectivity-map (CMap) predicted molecules that reverse the metabolic signature of cabozantinib-resistant AML cells and these molecules showed anti-leukmic activities

Leveraging large-scale small molecule perturbation libraries enables drug repositioning strategies to target specific pathways and gene regulatory networks [16]. To identify molecules capable of reversing metabolic reprogramming in cabozantinib-resistant cells, we used significant metabolism-related DEGs from cabozantinib-resistant and parental cells to query the Connectivity Map (CMap) transcriptomic database [17, 18]. This analysis predicted several compounds that could potentially counteract the metabolic reprogramming phenotypes of cabozantinib-resistant cells (Supplementary Tables S3–S4). Among them, two HSP90 inhibitors, radicicol (monorden) and tanespimycin (17-AAG), were identified as potential candidates for reversing the metabolic signature of Molm13-XR cells. Meanwhile, the PI3K inhibitor wortmannin and the mTOR inhibitor sirolimus were predicted to modulate the metabolic phenotypes of MV4-11-XR cells.

**Fig. 4:**
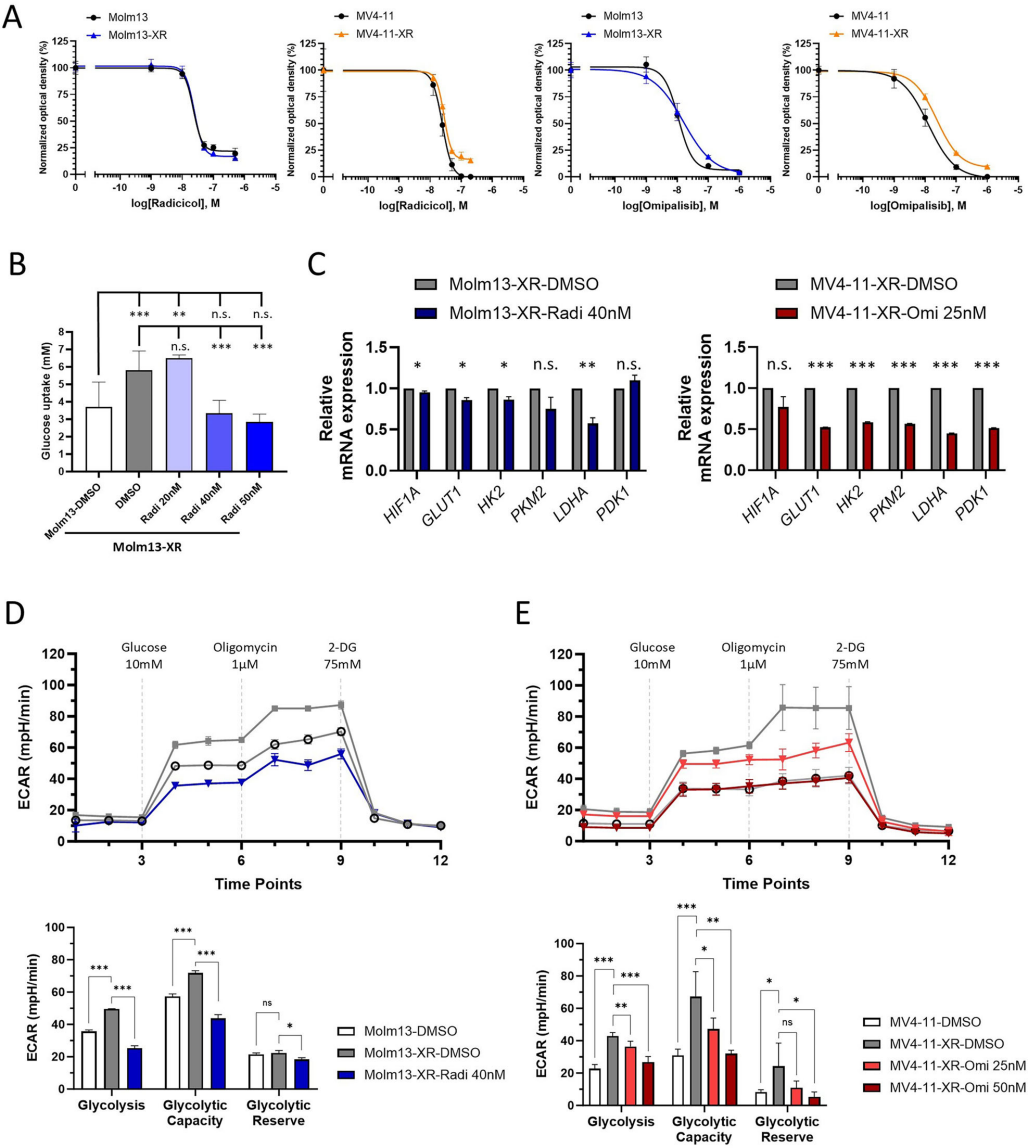
Assessment of the effects of CMap-predicted molecules in cabozantinib-resistant cells. **A** Metabolic activity assay of the indicated cells after treatment with radicicol or omipalisib for 72 h. **B** Glucose consumption of Molm13 and Molm13-XR cells treated with DMSO or radicicol (Radi) for 24 h. **C** RT-qPCR analyses of indicated genes in Molm13-XR (left panel) or MV4-11-XR (right panel) cells after DMSO, radicicol (Radi) or omipalisib (Omi) treatment for 24 h. 18 s rRNA was used for normalization. **D**, **E** Seahorse Glycolysis Stress Test evaluated basal glycolysis, glycolytic capacity, and glycolytic reserve after drug exposure or DMSO treatment for 24 h. Data are representative of two independent experiments each performed in triplicate. *p < 0.05, **p < 0.01, ***p < 0.001, compared with parental cells or DMSO group.

Afterward, we explored the metabolic regulatory effects of radicicol and omipalisib, a PI3K/mTOR dual inhibitor, in Molm13-XR and MV4-11-XR cells, respectively. Metabolic activity assays demonstrated that both radicicol and omipalisib exhibited sub-nanomolar inhibitory concentrations against Molm13-XR, MV4-11-XR, and their parental cells (Fig. 4A). Treatment of Molm13-XR cells with 40 nM radicicol for 24 h restored glucose uptake to levels observed in Molm13 cells (Fig. 4B), accompanied by decreased *LDHA* expression (Fig. 4C, left panel). Additionally, radicicol treatment inhibited the hyperactive glycolysis observed in Molm13-XR cells, as evidenced by reductions in basal glycolytic rate and glycolytic capacity (Fig. 4D). Similarly, treatment of MV4-11-XR cells with omipalisib reduced the expression of metabolism-related genes (Fig. 4C, right panel) and impaired their basal glycolytic rate and glycolytic capacity (Fig. 4E). These findings indicate that radicicol and omipalisib not only inhibit the growth of Molm13-XR and MV4-11-XR cells but also effectively reverse the enhanced glycolysis associated with cabozantinib resistance.

**Fig. 5:**
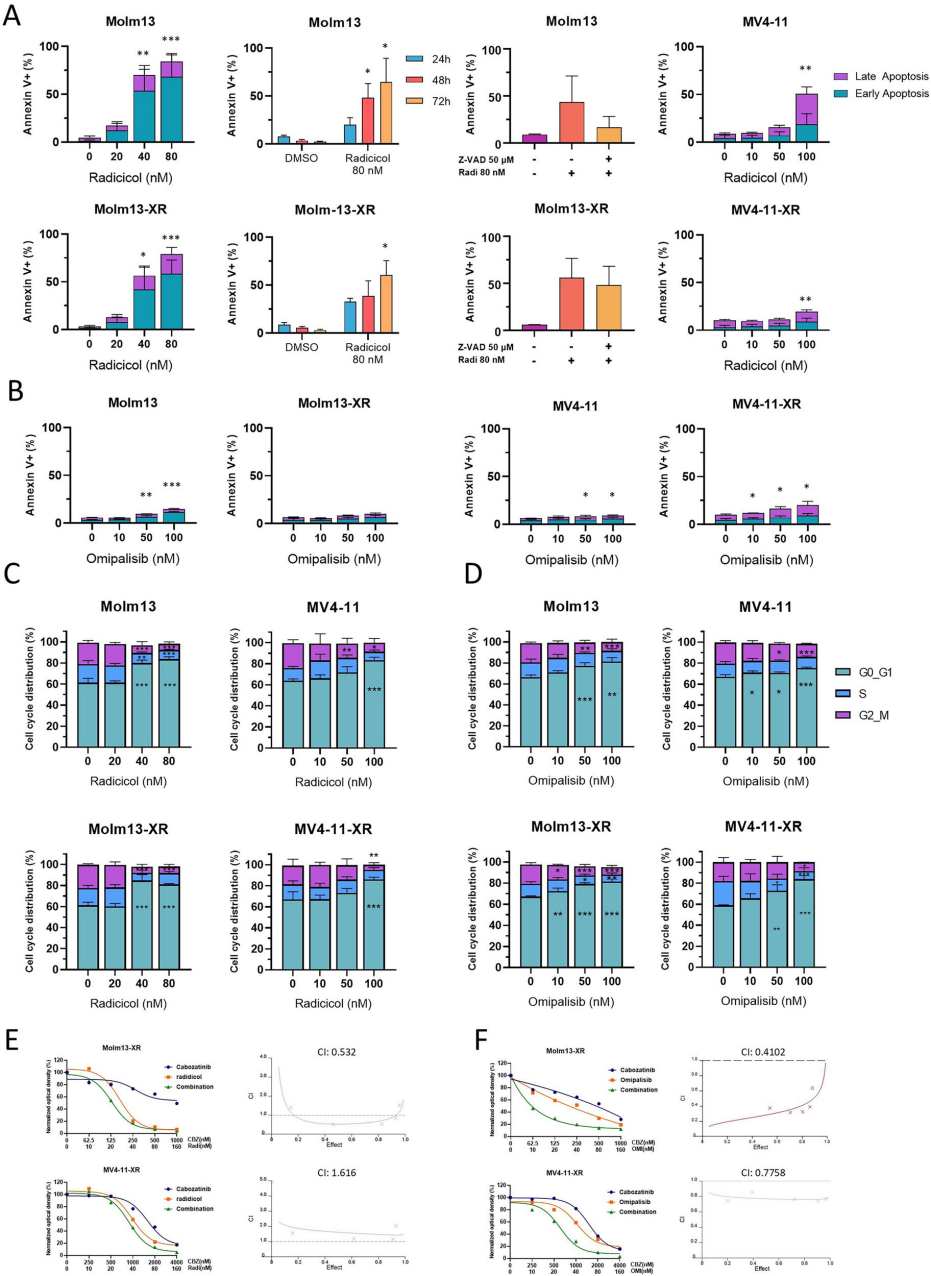
Evaluation of the anti-leukemic effects of radicicol and omipalisib in cabozantinib-resistant cells and their parental cells. **A**, **B** The apoptosis of indicated cells was evaluated by Annexin V and PI staining coupled with flow cytometry after 72 h drug exposure or indicated time points. **C**, **D** The cell cycle stage distribution of indicated cells was evaluated by PI staining coupled with flow cytometry after 24 h indicated drug exposure. **E**, **F** Cell viability of indicated cells evaluated by MTS assay after treatment with radicicol or omipalisib alone or in combination with cabozantinib for 72 h. Data are representative of two independent experiments each performed in triplicate. *p < 0.05, **p < 0.01, ***p < 0.001, compared with parental cells or DMSO group.

Next, we evaluated the anti-leukemic effects of radicicol and omipalisib. Radicicol induced significant apoptosis in Molm13 and Molm13-XR cells in a dose- and time-dependent manner (Fig. 5A), while omipalisib induced only limited apoptosis across all cell lines (Fig. 5B). Pretreatment with z-VAD could partially inhibit radicicol-induced apoptosis-dependent cell death, suggesting that apoptosis plays a role in radicicol-induced cell death (Fig. 5A). Moreover, both radicicol and omipalisib induced significant cell cycle arrest at G_0_/G_1_ phase in all tested cell lines (Fig. 5C–D). To determine whether radicicol and omipalisib could reverse cabozantinib resistance, Molm13-XR and MV4-11-XR cells were treated with either compound alone or in combination with cabozantinib. Co-treatment with radicicol and cabozantinib exhibited a synergistic effect in Molm13-XR cells but not in MV4-11-XR cells (Fig. 5E). Conversely, the combination of omipalisib and cabozantinib demonstrated synergistic effects in both Molm13-XR and MV4-11-XR cells (Fig. 5F).

## Discussion

In this study, we established two human cabozantinib-resistant AML cell lines, Molm13-XR and MV4-11-XR, derived from *FLT3*-ITD-positive Molm13 and MV4-11 cells, respectively. Both resistant cell lines exhibited cross-resistance to the FDA-approved sorafenib and quizartinib. Sanger sequencing identified the acquisition of the FLT3 D835Y mutation in both resistant cell lines, along with a novel *FLT3*¹^.^³ mutation in MV4-11-XR cells. Next, metabolic profiling revealed enhanced glycolysis and impaired oxidative phosphorylation as features of cabozantinib resistance. Using in silico drug prediction and experimental validation, we identified the HSP90 inhibitor radicicol and the PI3K/mTOR dual inhibitor omipalisib as potential therapeutic agents that reversed the metabolic reprogramming associated with resistance. Moreover, both inhibitors induced G_0_/G_1_ cell cycle arrest and exhibited anti-leukemic effects.

FLT3 is a key regulator of leukemic cell survival and proliferation, and FLT3 mutations play a major role in AML pathogenesis and therapy resistance. Sanger sequencing revealed that Molm13-XR and MV4-11-XR cells had substantially increased FLT3 D835Y burden, suggesting strong clonal selection under cabozantinib exposure. In addition to the D835Y mutation, we identified a novel *FLT3*¹^.^³ deletion in MV4-11-XR cells, spanning intron 19 to intron 20, which results in the complete loss of exon 20. Unlike previously reported FLT3 alterations, including internal tandem duplications (ITDs) and point mutations in the tyrosine kinase domain (TKD), *FLT3*¹^.^³ represents a large structural deletion that disrupts a critical functional region of the receptor. Exon 20 encodes part of tyrosine kinase domain 2 (TKD2), including the Asp-Phe-Gly (DFG) motif (aa 829-831) [19] and a portion of the activation loop (aa 829-858), both of which are essential for kinase activity and inhibitor binding, consistent with its cross-resistance to quizartinib and sorafenib. Our sequencing analysis suggested that the *FLT3*¹^.^³ mutant may come from recombination between homologous sequences flanking exon 20 (Supplementary Fig. S2C). Given that *FLT3* mutations typically arise from point mutations or duplications, this large deletion due to recombination highlights a distinct resistance mechanism that has not been previously described. This mutation, to our knowledge, is the first reported FLT3 alteration characterized by a large deletion encompassing exon 20. Further structural and biochemical validation is warranted to figure out the precise impact of this deletion on FLT3 kinase activity and drug resistance.

In addition to different patterns and burdens of FLT*3* mutations, we found that metabolic dysregulation is a hallmark of cabozantinib resistance. Resistant cells exhibited enhanced glycolysis and impaired oxidative phosphorylation, suggesting a shift toward a more glycolysis-dependent metabolic phenotype. Hyperactive glycolysis has been observed in various cancer types upon resistance development [20, 21]. Sorafenib-resistant BaF3/ITD and MV4-11 cells exhibited impaired mitochondrial function and upregulated glycolysis [22], accompanied by reduced glucose flux into the oxidative arm of the pentose phosphate pathway [23]. Similarly, imatinib-resistant *BCR-ABL*-transduced Ba/F3 and LAMA-84 cells displayed increased glycolytic rates and hyperactivation of HIF-1α [24], a key regulator of glycolysis. Comparable findings were reported in nilotinib- and dasatinib-resistant K562 cells [25]. While these studies established an association between metabolic reprogramming and drug resistance, the precise consequences of TKI resistance on metabolic alterations remain unclear. In this study, the 32D cells expressing *FLT3*-ITD/ D835Y were found to be cabozantinib-resistant and displayed reduced glycolytic activity and impaired oxidative phosphorylation, and these were inconsistent with the hyperactive glycolytic activities of Molm13-XR and MV4-11-XR cells. This discrepancy may be due to genetic backgrounds and species differences. Therefore, no further experiments were conducted on 32D-ITD D835Y cells using agents that regulate metabolism. This is the first finding that secondary *FLT3* mutations could directly induce metabolic alterations in leukemia cells. Further studies are needed to determine the molecular mechanism underlying FLT3-TKD mutations, metabolic reprogramming, and TKI-resistance in AML cells.

Interestingly, we found that in Molm13-XR cells, despite the upregulation of mitochondrial biogenesis–associated genes (including *PPARGC1B*, *NRF1*, and *TFAM*), mitochondrial mass was reduced compared to Molm13 cells. Previous research has indicated similar phenomenon that the expression of the transcriptional coactivator PGC-1α is positively correlated with nuclear DNA-encoded mitochondrial genes, but negatively correlated with mitochondrial DNA-derived transcripts and mitochondrial content markers, suggesting that elevated expression of mitochondrial biosynthesis genes may coexist with decreased mitochondrial mass or mitochondrial DNA content, possibly influenced by factors such as cell proliferation, tissue-specific mitochondrial gene expression, and DNA replication regulation [26]. Furthermore, a study on Myc-regulated mitochondrial biosynthesis also showed that increased expression of mitochondrial biosynthesis genes may not be accompanied by a corresponding increase in mitochondrial DNA replication or mitochondrial mass, because factors such as TFAM control mitochondrial DNA copy number and mitochondrial transcription [27]. Therefore, the paradoxical phenomenon of increased expression of mitochondrial biosynthetic genes and decreased mitochondrial mass or DNA content was recorded in specific tissues and environments, reflecting a complex regulatory mechanism that goes beyond simple transcriptional activation. Even so, this study revealed that the OCR and mitochondria mass of both cabozantinib-resistant cell lines MV4-11-XR and Molm13-XR had consistent results, supporting that the metabolic reprogramming—combining glycolytic activation with maladaptive mitochondrial signaling—forms a multifaceted resistance mechanism in AML to cabozantinib.

Through in silico drug screening and functional validation, we identified HSP90 inhibitor radicicol and PI3K/mTOR dual inhibitor omipalisib as potential candidates for reversing metabolic reprogramming in resistant cells. HSP90 is a molecular chaperone that stabilizes and maintains the function of multiple oncogenic proteins, including those regulating the PI3K/AKT/mTOR signaling cascade—a pathway central to cancer cell survival, proliferation, and metabolism [28, 29]. Furthermore, hyperactivation of the PI3K/AKT/mTOR pathway is a well-documented mechanism of resistance to tyrosine kinase inhibitors (TKIs) in several malignancies, including Ph⁺ acute lymphoblastic leukemia (ALL) treated with ABL kinase inhibitors [30] and sorafenib-resistance in AML [31, 32]. Inhibition of HSP90 has been shown to suppress PI3K/AKT/mTOR signaling and induce cell death in diverse cancers such as Burkitt lymphoma and lung cancer [33, 34]. In the same way, targeting HSP90 or the PI3K/AKT/mTOR pathway overcomes TKI resistance in breast and lung cancers [35–37]. Consistent with these findings, our study observed increased PI3K/AKT/mTOR activity in cabozantinib-resistant AML cells, and the inhibition of HSP90 not only reverses cabozantinib resistance but also mitigates the enhanced glycolytic metabolism in resistant cells. Similarly, omipalisib, by directly targeting PI3K/mTOR signaling, significantly reduced glycolytic capacity in MV4-11-XR cells and impaired the expression of glycolytic-related genes, which expands our recent finding that omipalisib exerts anti-leukemic effects in OCI-AML3 and THP-1 cell lines by suppressing serine biosynthesis, glutathione metabolism, and mitochondrial biogenesis [38]. Collectively, our current work supports a mechanistic link between HSP90, PI3K/mTOR signaling, and TKI resistance in FLT3-mutant AML and demonstrated reversing metabolic changes as one of the mechanisms to mitigate TKI resistance by targeting the HSP90-PI3K-mTOR pathway.

We further demonstrated the anti-leukemic effects of radicicol and omipalisib across both resistant cell lines. Notably, synergistic effects were observed when combining omipalisib with cabozantinib to treat Molm13-XR and MV4-11-XR, suggesting its ability to reverse TKI resistance, expanding on previous findings of its application in sensitizing cancer cells to chemotherapy and radiotherapy [39, 40]. Although our study was performed only on cell line models, these findings provide a strong rationale for further validation in patient-derived samples or in vivo mouse models to better reflect the clinical situations. Evaluating the antileukemic activity and metabolic modulation of omipalisib or HSP90 inhibition in FLT3-mutant or cabozantinib-resistant AML xenograft models would help determine the translational potential of our findings and assess their therapeutic relevance. A clinical trial assessing omipalisib in patients with idiopathic pulmonary fibrosis reported a maximum observed concentration (Cmax) of 170 nM at a dose of 2.0 mg twice daily [41], which far exceeds the anti-leukemic dose used in our study. These findings support the feasibility of omipalisib as a potential therapeutic strategy for AML patients with sorafenib-, quizartinib-, and cabozantinib resistance.

## Conclusion

Overall, we established two human cabozantinib-resistant myeloid leukemia cell lines, Molm13-XR and MV4-11-XR cells. Both resistant cell lines exhibited cross-resistance to the FDA-approved sorafenib and quizartinib. In addition to characterizing these two cells, we provide critical insights into the mechanisms of cabozantinib resistance and unravel metabolic dysregulation as a key feature. Finally, we demonstrate that omipalisib and radicicol can effectively target the metabolic vulnerability of drug-resistant cells, reverse enhanced glycolysis, and restore drug sensitivity. These findings support the rationale for targeting metabolic pathways as a novel strategy to mitigate TKI resistance. Further validation in patient-derived models is warranted to translate these findings into clinical applications for AML treatment.

## Materials and methods

### Cell lines

Molm13 and MV4-11 cells were obtained, authenticated, and maintained in proper medium and conditions as previously described [8]. To establish the cabozantinib-resistant cell lines, parental Molm-13 and MV4-11 cells were initially cultured in RPMI1640 containing 10% FBS with cabozantinib at a concentration of their IC_50_ (39.3 nM and 0.3 nM, respectively). Once the growth of the cells resumed, the dose of cabozantinib was doubled. The process was repeated until the cells could no longer grow as fast as they had with the previous dose. The IC_50_ value of cabozantinib for these two cells was 602.0 nM and 6435 nM, respectively, as assessed by MTS cytotoxicity assay. These two cells were named as Molm13-XR and MV4-11-XR cells, respectively. Overall, it took approximately five months to establish Molm13-XR and MV4-11-XR cells. Since then, Molm13-XR and MV4-11-XR cells were continuously cultured in a medium without cabozantinib, and maintain their drug resistance. These cell lines were authenticated (16-Markers STR) by Food Industry Research and Development Institute, Hsinchu, Taiwan. The genetic profiles of these cell lines were identical to the reported genetic profiles.

### Cell viability assay, trypan blue counting, and colony-forming assay

Cabozantinib, sorafenib, quizartinib, and gliteritinib were purchased from Selleckchem Company (Houston, TX, USA). The cytotoxic effects of these compounds on various cells were evaluated by Colorimetric CellTiter 96 Aqueous One Solution Cell Proliferation Assay (MTS assay) (Promega, Madison, WI) according to the manufacturer’s instructions as previously mentioned [10], after 72 h exposure to the drug. The IC_50_ values and the synergy of drugs, represented by the combination index (CI), were estimated by the CalcuSyn software with the Chou-Talalay method [42].

The cell proliferation assay was estimated with the PI (propidium iodide) exclusion method combined with flow cytometry every day to assess the growth kinetics of cabozantinib-resistant cells and their parentals. For evaluating the colony formation ability of leukemic cell lines, cells were cultured in MethoCult™ H4434 Classic medium (STEMCELL, Vancouver, Canada) for 14 days according to the manufacturer’s instructions. The colony formation rate was evaluated as the number of colonies divided by the seeded cell number. One colony was defined as a cluster containing more than 50 cells. The number of colonies for each cell line was manually counted under a microscope according to this criterion.

### Immunoblotting

As previously described [43], the cell lysates were subjected to 10% polyacrylamide gel and transferred to a polyvinylidene fluoride (PVDF) membrane. After blocking, membranes were incubated with the indicated primary antibodies (Supplementary Table S5) and subsequently reacted with horseradish peroxidase-conjugated secondary antibodies (Cell Signaling Technology, Beverly, MA, USA). Finally, the chemiluminescence signals were developed by Western Lightning Plus-ECL (Perkin Elmer, Waltham, MA, USA) and captured by LAS 4000 (Fujifilm, Tokyo, Japan).

### Cellular bioenergetic analysis

Cellular mitochondrial and glycolytic functions were assessed by analyzing oxygen consumption rate (OCR) and extracellular acidification rate (ECAR) from the XFe24 extracellular flux analyzer (Agilent Technologies/Seahorse Bioscience, Billerica, MA, USA) according to the manufacturer’s protocol. After 24 h prior exposure to the drug, cells were then seeded into each well of a 24-well microplate precoated with Cell-Tak Cell and Tissue Adhesive (Corning, Corning, NY, USA) as previously described [43]. Mito Stress Test kit (Agilent Technologies/Seahorse Bioscience) was used to measure mitochondrial respiration. After measuring baseline respiration, oligomycin A, FCCP, rotenone, and antimycin A were added sequentially into the microplate to detect the oxygen consumption rate. Glycolysis Stress Test kit (Agilent Technologies/Seahorse Bioscience) was used to assess glycolytic activity. After 1 h of glucose starvation, glucose, oligomycin, and 2-DG were added sequentially to measure the glycolytic activity. The results were analyzed by Seahorse XFe24 wave software 2.2 (Agilent Technologies/Seahorse Bioscience).

### Extracellular glucose consumption and lactate production

For the measurement of extracellular glucose consumption, a total of 1 × 10^5^ cells in 500 μL of complete medium were seeded in 24-well plates overnight. Supernatants were harvested by centrifugation (500 × *g*, 5 min, 4 °C) and the concentration of glucose was measured by Accu-Chek Performa (Roche, Basel, Switzerland) according to the manufacturer’s instructions. The glucose consumption was calculated as the difference between the glucose concentration of the cultured supernatant and the cell-free medium.

To measure the lactate production from cells, the Lactate Colorimetric/Fluorometric Assay Kit (BioVision, MA, USA) was used according to the manufacturer’s instructions. Briefly, a total of 2 × 10^5^ cells were resuspended in 500 μL serum-free medium overnight before the collection of supernatants. After transferring supernatants through a 10 kDa filter tube (Millipore, MA, USA), the eluates were collected by centrifugation (700 × *g*, 10 min, 4 °C). Each reaction in a 96-well plate contained 2 μL of eluates or serially diluted standards, 48 μL of lactate buffer, and 50 μL of enzyme premix. After shaking at room temperature for 30 min, the O.D. 570 nm was measured using VersaMax M5 (Molecular Devices, CA, USA), and the concentration of lactate was calculated according to the standard curve.

### Flow cytometry

For cell cycle analysis, after 24 h of drug exposure, the cells were fixed in 70% ethanol and stained with propidium iodide (PI; BD Biosciences, Franklin Lakes, NJ, USA). Cell cycle distribution was analyzed using FlowJo^TM^ software v10.4.

For apoptosis analysis, cells were treated with the indicated drugs for 72 h, and the cell apoptosis was assessed by FITC Annexin-V Apoptosis Detection Kit I (BD Biosciences). FlowJo^TM^ software v10.4 was used to determine the relative frequency between viable (AV − /PI − ), early (AV + /PI − ), and late (AV + /PI + ) apoptotic cells. The caspase inhibitor Z-VAD(OMe)-FMK (#14463, Cayman Chemical; Ann Arbor, MI, USA) was added 30 min before drug exposure, in a final concentration of 50 μM.

To assess reactive oxygen species (ROS) content and mitochondrial membrane potential, cells were stained with 10 μM DCFH-DA (2’,7’-dichlorodihydrofluorescein diacetate dye; Sigma) and 400 nM TMRE (Invitrogen), respectively, according to the manufacturer’s instructions, and the fluorescence intensities were measured with a CYTOFLEX^TM^ Flow Cytometer (Beckman Coulter).

### Statistical analysis

The statistical differences between measurements were assessed by the two-sided Student’s *t*-test. Error bars in the graphs indicate the means ± SD in triplicate or means ± SEM of at least three independent experiments. The *p-value* less than 0.05 were considered statistically significant (*p < 0.05; **p < 0.01; ***p < 0.001).

## Supplementary information

Supplementary materials

Original Data Files

Related Manuscript File

## Data Availability

The transcriptome data presented in this study are available in the Gene Expression Omnibus (GEO) database (ID: GSE234724).
