# Supplementary material for: Modulating metabolic signatures to mitigate cabozantinib resistance in FLT3-ITD acute myeloid leukemia cell models
Source: Cell Death Discov. 2026 Feb 17;12:98. doi: 10.1038/s41420-026-02957-8 (PMC12920922; doi:10.1038/s41420-026-02957-8)
Supplement: Supplementary file 1 — Supplementary materials [file 41420_2026_2957_MOESM1_ESM.docx]

**SUPPLEMENTARY MATERIALS**

**Supplementary Figure S1.** (**A**) Representative images of colonies from the indicated cell lines. (**B**) Phosphoarray immunoblotting images and the quantification of the protein phosphorylation level in Molm13 or Molm13-XR cells (**C**) Sanger sequencing analysis of the *FLT3* gene revealed the emergence of a new FLT3 mutation D835Y (c.2503G>T) mutation in Molm13-XR and MV4-11-XR cells. (**D**) The variant allele frequency (VAF) of *FLT3* c.2503G>T mutation in the indicated cell lines was determined by pyrosequencing.


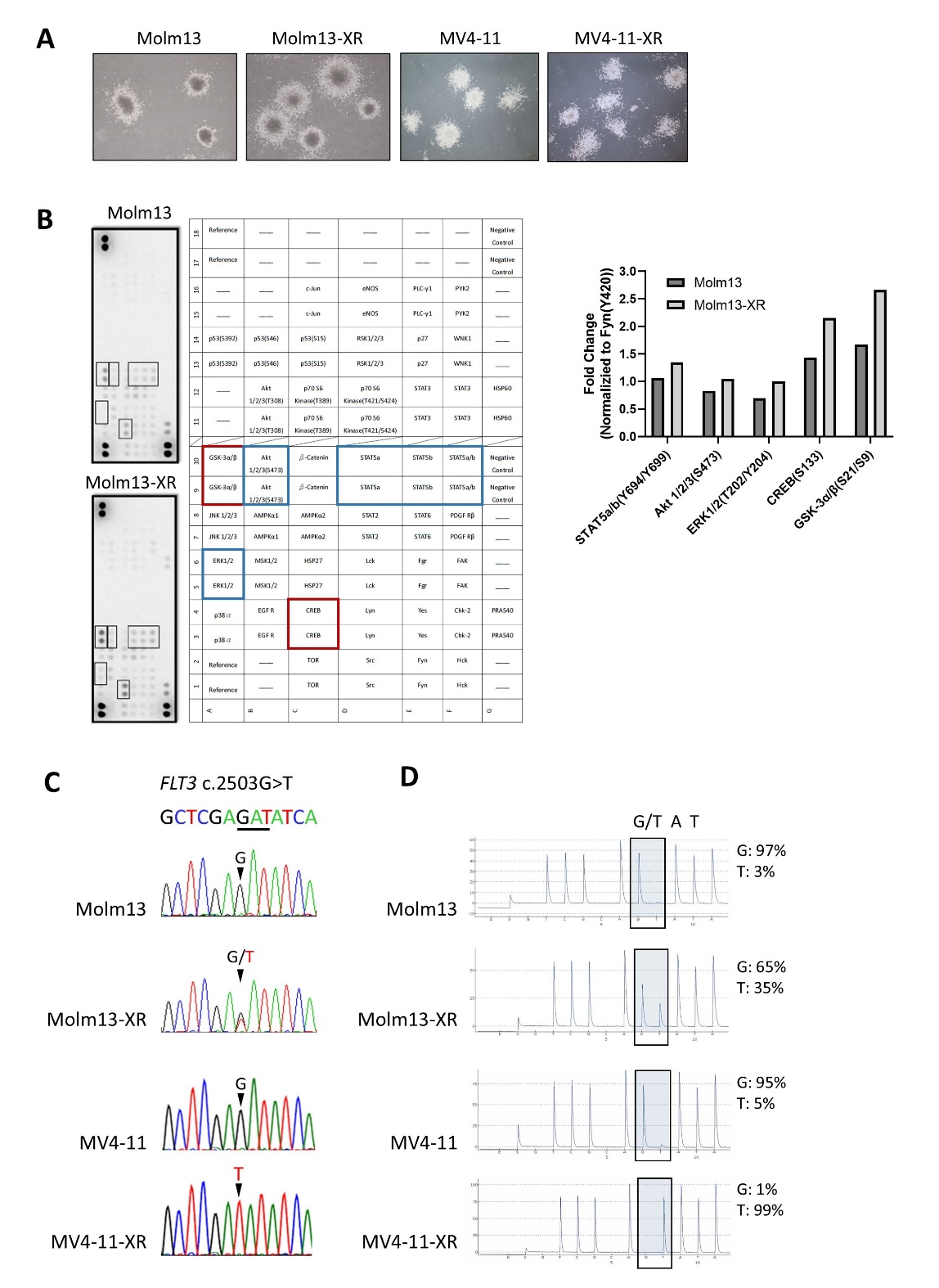


**Supplementary Figure S2.** (**A**) DNA capillary electrophoresis shows the frequency of full-length *FLT3* allele and Δexon20 allele in MV4-11 and MV4-11-XR cells. (**B**) Exon-intron junction sequence in MV4-11-XR cells. (**C**) Schematic representation illustrates the Δexon20 in MV4-11-XR cells. (**D**) Gel electrophoresis image to show two different products after amplifying *FLT3* region from cDNA of MV4-11-XR cells. (**E**) Sanger sequencing chromatograms demonstrated the (a) wild-type *FLT3* sequences in MV4-11, (b) *FLT3* D835Y (c.2503G>T) mutation, and (c) Δexon20 in MV4-11-XR cells, and. *p < 0.05, **p < 0.01, ***p < 0.001.


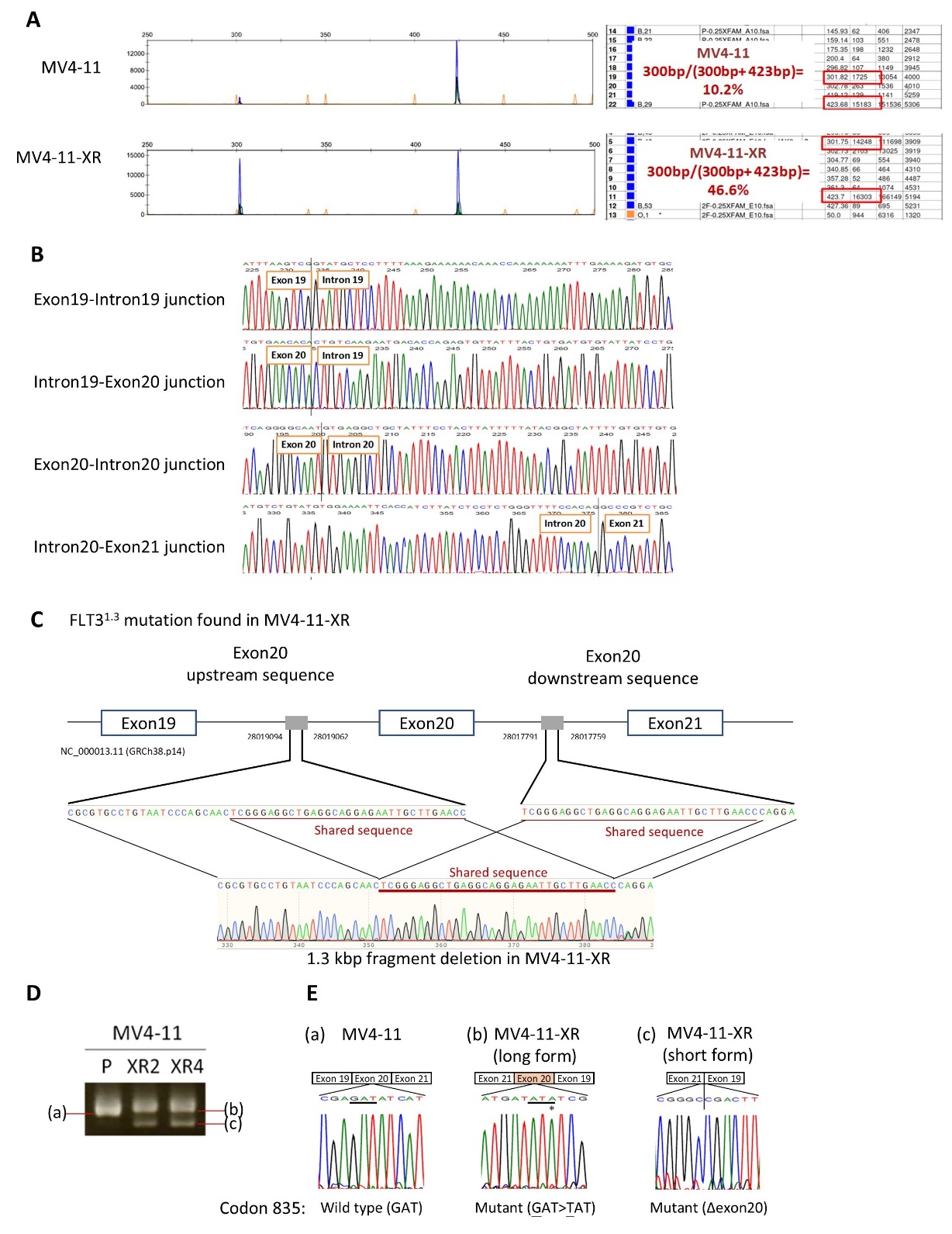


**Supplementary Figure S3.** (**A**) Cell viability of 32D cells harboring various *FLT3* mutations was evaluated by MTS assay after cabozantinib treatment for 72 h. (**B-C**) Seahorse Cell Mito Stress Test and Glycolysis Stress Test were performed to measure various metabolic parameters in the indicated cells. Data are representative of two independent experiments each performed in triplicate. *p < 0.05, **p < 0.01, ***p < 0.001, compared with parental cells.


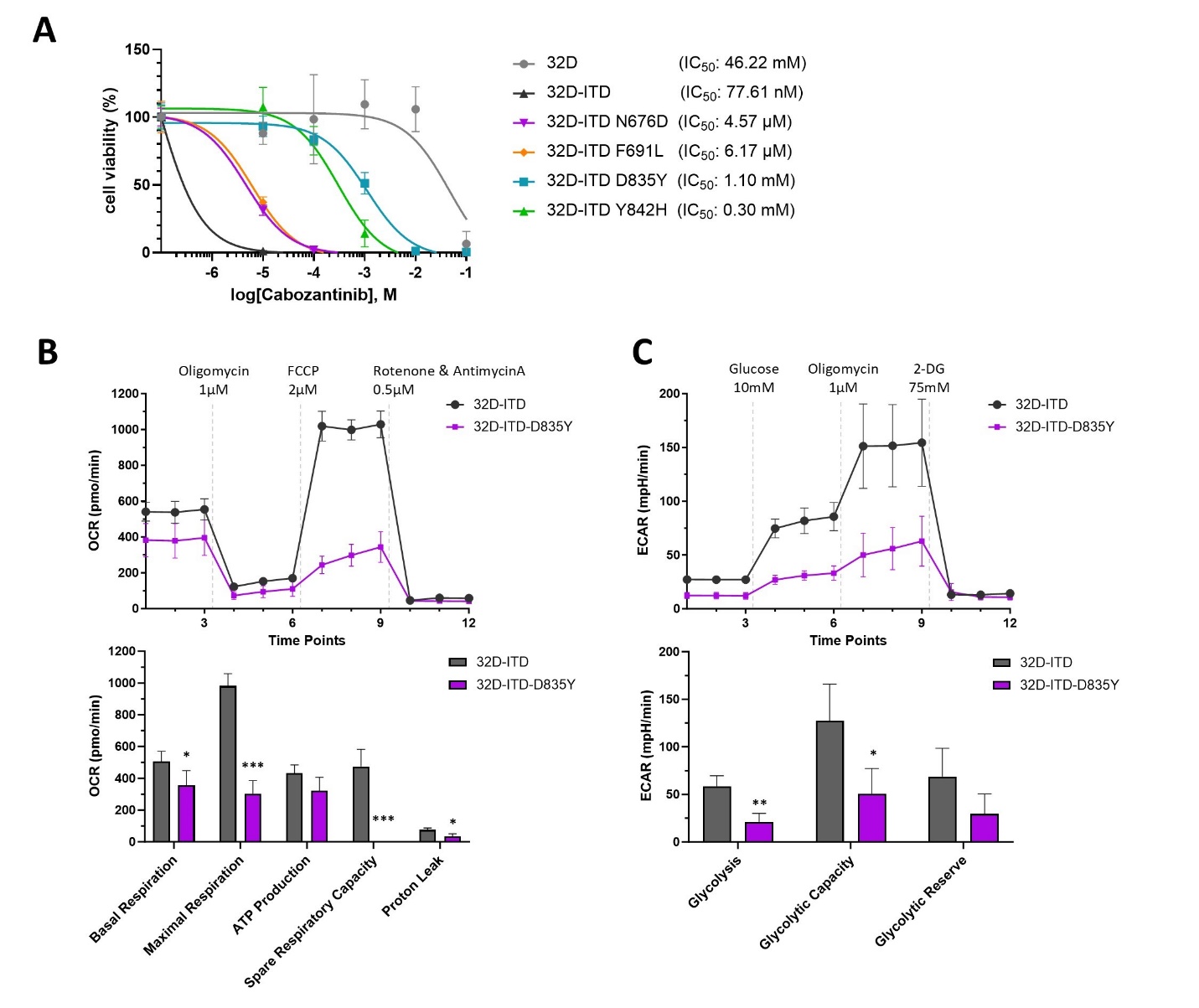


**Supplementary Table S1.** Positive percentage (%) of various surface markers on cabozantinib-resistant cells and their parentals.

| Surface marker | Molm13 | Molm13-XR | MV4-11 | MV4-11-XR |
| --- | --- | --- | --- | --- |
| HLA-DR | 0.7 | 0.2 | 2.9 | 4.1 |
| CD34 | 3.3 | 12.7 | 0.7 | 1.4 |
| CD13 | 25.1 | 12.1 | 52.5 | 29.0 |
| CD33 | 100.0 | 100.0 | 99.9 | 100.0 |
| CD14 | 0.2 | 0.5 | 0.3 | 0.6 |
| CD11b | 20.2 | 10.0 | 3.0 | 2.4 |
| CD15 | 100.0 | 100.0 | 100.0 | 100.0 |
| CD19 | 0.1 | 0.1 | 0.4 | 0.8 |
| CD20 | ND | ND | 0.3 | 0.1 |
| CD7 | 0.3 | 0.1 | 0.3 | 0.1 |
| CD2 | 0.2 | 0.2 | 2.3 | 0.7 |
| CD5 | ND | ND | 0.2 | 0.1 |
| CD3 | ND | ND | 0.3 | 1.0 |
| CD56 | 93.8 | 99.9 | 4.7 | 6.8 |
| CD16 | 0.2 | 0.3 | 40.4 | 69.8 |
| CD38 | 88.1 | 99.0 | 0.1 | 0.1 |
| CD235 (GPA) | 0.2 | 0.5 | 0.9 | 1.6 |
| CD41 | 0.4 | 1.5 | 4.7 | 11.6 |
| CD123 | 58.7 | 46.2 | ND | ND |
| CD117 | 0.2 | 0.3 | ND | ND |

**Supplementary Table S2.** List of metabolic genes showing significant changes in Molm13-XR vs. Molm13, or MV4-11-XR vs. MV4-11.

| Molm13-XR vs. Molm13 | | |  | MV4-11-XR vs. MV4-11 | | |
| --- | --- | --- | --- | --- | --- | --- |
| Gene name | Adjusted  p-value | Fold change |  | Gene name | Adjusted  p-value | Fold change |
| ABHD14A-ACY1 | 1.79E-07 | 58.8349 |  | AGMAT | 2.66E-03 | 2.9095 |
| ACAA2 | 2.84E-04 | 2.1061 |  | AGPAT4 | 1.24E-11 | 6.3514 |
| ACSL1 | 4.57E-05 | 2.1349 |  | ALDH5A1 | 6.60E-03 | 2.0885 |
| ACSS1 | 1.00E-02 | 1.8611 |  | ASNS | 3.57E-11 | 3.5568 |
| AGMAT | 2.01E-06 | 2.6923 |  | ASS1 | 6.40E-04 | 2.2567 |
| AK1 | 3.16E-02 | 3.3550 |  | BCAT1 | 4.76E-09 | 3.1185 |
| AK4 | 0.00E+00 | 18.0232 |  | BCAT2 | 1.47E-02 | 2.0613 |
| ALAS1 | 1.95E-06 | 2.3229 |  | CBS | 7.48E-13 | 5.1013 |
| ALDH1A3 | 0.00E+00 | 41.5863 |  | CHAC1 | 1.75E-13 | 6.5422 |
| ALOX12 | 4.60E-03 | 28.0708 |  | CHPT1 | 1.01E-02 | 2.6721 |
| AMACR | 3.45E-02 | 2.1416 |  | DGAT2 | 2.58E-04 | 2.4850 |
| AOC1 | 0.00E+00 | 15.4145 |  | DGKH | 4.88E-07 | 3.2151 |
| ARSB | 1.61E-04 | 2.1061 |  | GCLM | 1.05E-06 | 2.7616 |
| ASL | 1.12E-04 | 2.4798 |  | HMOX1 | 9.99E-16 | 5.9232 |
| ATP6V0E1 | 8.95E-03 | 1.8551 |  | HSD11B1 | 0.00E+00 | 24.5817 |
| B3GNT5 | 0.00E+00 | 10.0285 |  | LPCAT2 | 0.00E+00 | 7.0838 |
| B4GALT5 | 5.92E-03 | 1.8729 |  | ME1 | 0.00E+00 | 12.3253 |
| B4GALT6 | 1.73E-02 | 24.2036 |  | NADK2 | 5.45E-11 | 3.4848 |
| CD38 | 0.00E+00 | 11.7011 |  | NAMPT | 1.09E-02 | 2.0447 |
| CERS1 | 3.61E-03 | 3.3232 |  | NDST1 | 2.22E-11 | 7.1807 |
| CHPF | 4.16E-13 | 5.9748 |  | NDUFS6 | 4.28E-05 | 2.4641 |
| CHPT1 | 3.63E-04 | 2.3278 |  | NDUFV2 | 5.46E-03 | 2.0883 |
| CKMT1B | 8.88E-16 | 18.0105 |  | PCK2 | 1.72E-06 | 2.8380 |
| CTPS2 | 3.33E-16 | 13.6939 |  | PHGDH | 5.82E-11 | 3.4683 |
| CYP27B1 | 2.49E-03 | 3.0088 |  | POLR3G | 6.13E-05 | 2.6609 |
| DHRS3 | 1.10E-13 | 43.0498 |  | PSAT1 | 7.18E-08 | 2.9097 |
| DPYD | 0.00E+00 | 13.8404 |  | RDH10 | 0.00E+00 | 9.4628 |
| GAA | 5.17E-04 | 2.0952 |  | SDHA | 6.05E-03 | 2.0924 |
| GALM | 3.25E-07 | 2.5518 |  | SLC27A5 | 2.51E-02 | 2.2451 |
| GALNT10 | 1.80E-02 | 1.8699 |  | SPHK1 | 1.40E-03 | 3.0560 |
| GALNT2 | 1.81E-05 | 2.4793 |  | ABHD14A-ACY1 | 4.79E-09 | 0.0139 |
| GBGT1 | 0.00E+00 | 8.0490 |  | ACOT1 | 4.48E-04 | 0.0286 |
| GCH1 | 7.72E-03 | 3.3931 |  | ACOT2 | 0.00E+00 | 0.0044 |
| GGT1 | 1.38E-02 | 1.9768 |  | AGK | 0.00E+00 | 0.0042 |
| GGT5 | 3.72E-14 | 18.9079 |  | AKR1B1 | 1.20E-06 | 0.3709 |
| GUSB | 7.56E-03 | 1.9046 |  | ALDH3A2 | 7.40E-07 | 0.3271 |
| HACD1 | 3.28E-03 | 2.9700 |  | ALDH3B1 | 1.10E-06 | 0.3354 |
| HEXB | 2.46E-03 | 1.9517 |  | ANPEP | 4.02E-10 | 0.0124 |
| HSD11B1 | 0.00E+00 | 7.2058 |  | B4GALT6 | 0.00E+00 | 0.0053 |
| INPP1 | 6.00E-15 | 7.3272 |  | BDH2 | 3.76E-02 | 0.4701 |
| LDHAL6A | 4.11E-04 | 7.6379 |  | BST1 | 2.91E-04 | 0.2021 |
| MAN1A1 | 1.65E-13 | 105.4161 |  | CBR1 | 0.00E+00 | 0.0016 |
| MGAT4A | 0.00E+00 | 12.9777 |  | CBR3 | 6.58E-04 | 0.0644 |
| MGAT4B | 6.03E-05 | 2.2132 |  | CMBL | 2.24E-02 | 0.1417 |
| MGLL | 1.65E-13 | 105.4161 |  | CSGALNACT1 | 1.60E-03 | 0.2448 |
| MINPP1 | 7.42E-13 | 5.8088 |  | CTPS2 | 0.00E+00 | 0.0019 |
| NAGA | 7.57E-04 | 1.9968 |  | ELOVL6 | 4.02E-10 | 0.0124 |
| NDST1 | 6.91E-09 | 2.6638 |  | GAA | 1.07E-02 | 0.4792 |
| NDUFA2 | 3.74E-02 | 1.7978 |  | GALNT4 | 2.48E-05 | 0.0228 |
| NT5C3B | 3.25E-14 | 111.2170 |  | GATM | 6.55E-03 | 0.0372 |
| OGDHL | 7.45E-04 | 3.7481 |  | GSTZ1 | 1.74E-02 | 0.4762 |
| PCBD2 | 2.48E-03 | 2.5193 |  | H6PD | 2.70E-09 | 0.0897 |
| PLA2G4B | 3.94E-03 | 4.5136 |  | HAL | 6.17E-11 | 0.2371 |
| PLD4 | 1.87E-07 | 2.5139 |  | HK3 | 7.55E-05 | 0.2456 |
| PTGIS | 8.81E-03 | 26.1759 |  | HSD17B4 | 0.00E+00 | 0.0286 |
| SAT1 | 2.34E-02 | 1.8753 |  | HYAL3 | 1.07E-03 | 0.4000 |
| TYMP | 0.00E+00 | 4.4051 |  | HYI | 4.61E-03 | 0.1054 |
| UGDH | 2.61E-06 | 3.1097 |  | INPP1 | 5.55E-16 | 0.0075 |
| UQCRQ | 1.54E-03 | 1.9493 |  | INPP5F | 1.91E-02 | 0.4746 |
| BST1 | 4.57E-14 | 0.0227 |  | KYNU | 2.09E-13 | 0.2352 |
| GCNT2 | 1.80E-03 | 0.0719 |  | LPIN3 | 2.18E-10 | 0.0424 |
| MGAT3 | 4.49E-02 | 0.0980 |  | MBOAT1 | 1.26E-06 | 0.2758 |
| GNE | 0.00E+00 | 0.1508 |  | ME3 | 1.28E-04 | 0.3095 |
| CPS1 | 9.51E-09 | 0.2056 |  | MTMR1 | 0.00E+00 | 0.0201 |
| CMPK2 | 8.98E-04 | 0.2096 |  | NMNAT3 | 2.58E-04 | 0.2157 |
| AGK | 2.07E-08 | 0.3444 |  | NT5C3B | 4.57E-04 | 0.0287 |
| NTPCR | 3.79E-04 | 0.3788 |  | NUDT12 | 6.55E-03 | 0.0372 |
| HSD17B12 | 6.85E-05 | 0.4419 |  | OAT | 0.00E+00 | 0.0012 |
| GCLC | 4.37E-04 | 0.4669 |  | PGM1 | 0.00E+00 | 0.0029 |
| COQ3 | 8.63E-04 | 0.4731 |  | PIP5K1A | 0.00E+00 | 0.0528 |
| KYNU | 6.91E-03 | 0.4755 |  | PLB1 | 1.16E-03 | 0.3890 |
| TBXAS1 | 5.45E-03 | 0.4952 |  | PLCB4 | 2.96E-03 | 0.1611 |
| PGM3 | 1.40E-02 | 0.5269 |  | PLCG1 | 5.37E-03 | 0.4367 |
| MTM1 | 1.31E-02 | 0.5317 |  | POMGNT2 | 2.97E-12 | 0.0101 |
|  |  |  |  | RIMKLA | 1.24E-09 | 0.0130 |
|  |  |  |  | RIMKLB | 1.14E-10 | 0.0280 |
|  |  |  |  | SPTLC2 | 3.11E-03 | 0.4742 |
|  |  |  |  | SUOX | 1.65E-04 | 0.3566 |
|  |  |  |  | TYMP | 1.57E-02 | 0.4826 |
|  |  |  |  | UAP1L1 | 2.56E-04 | 0.2156 |
|  |  |  |  | UGCG | 0.00E+00 | 0.0845 |

**Supplementary Table S3.** List of candidate molecules from the connectivity map queried by metabolic-related differentially expressed genes (DEGs) by Molm13-XR vs Molm13

| **Rank** | **Cmap molecules** | **Mean**  **Score** | **No of**  **instances** | **Enrichment** | **P value** | **Specificity** | **Percent non-null** |
| --- | --- | --- | --- | --- | --- | --- | --- |
| 1 | lomustine | 0.778 | 4 | 0.971 | 0 | 0.0118 | 100 |
| 2 | Monorden (radicicol) | 0.360 | 22 | 0.518 | 0 | 0.0321 | 63 |
| 3 | Tanespimycin (17-AAG) | 0.382 | 62 | 0.499 | 0 | 0.0725 | 62 |
| 4 | chloroquine | 0.615 | 4 | 0.885 | 0.00018 | 0 | 100 |
| 5 | azaperone | 0.569 | 4 | 0.845 | 0.00088 | 0 | 100 |
| 6 | amodiaquine | -0.616 | 4 | -0.833 | 0.00141 | 0.0066 | 100 |
| 8 | trimethobenzamide | 0.319 | 5 | 0.747 | 0.00236 | 0.0184 | 60 |
| 9 | norethisterone | -0.567 | 4 | -0.808 | 0.00265 | 0 | 75 |
| 10 | sulfamonomethoxine | 0.469 | 4 | 0.798 | 0.00318 | 0.0186 | 75 |
| 11 | spectinomycin | -0.454 | 4 | -0.797 | 0.00334 | 0 | 75 |
| 12 | calycanthine | 0.278 | 4 | 0.791 | 0.00368 | 0.0177 | 50 |
| 14 | minaprine | 0.327 | 5 | 0.698 | 0.00605 | 0 | 60 |
| 18 | pizotifen | -0.496 | 4 | -0.753 | 0.00758 | 0.0086 | 75 |
| 19 | gliclazide | -0.277 | 4 | -0.751 | 0.00768 | 0.02 | 50 |
| 21 | 0317956-0000 | 0.247 | 8 | 0.545 | 0.00937 | 0.0148 | 50 |
| 22 | pridinol | -0.24 | 4 | -0.736 | 0.00961 | 0.0054 | 50 |
| 23 | fluoxetine | 0.397 | 4 | 0.725 | 0.01148 | 0.0156 | 75 |
| 24 | flurbiprofen | -0.476 | 5 | -0.657 | 0.01162 | 0.024 | 80 |
| 25 | delsoline | -0.355 | 4 | -0.724 | 0.01192 | 0.0226 | 50 |
| 27 | hexestrol | -0.356 | 4 | -0.715 | 0.01339 | 0.0511 | 50 |
| 29 | Prestwick-984 | 0.225 | 4 | 0.709 | 0.0148 | 0.0303 | 50 |
| 31 | pivampicillin | -0.286 | 4 | -0.703 | 0.01611 | 0.0214 | 50 |
| 32 | xylazine | -0.277 | 4 | -0.702 | 0.01643 | 0.0105 | 50 |
| 33 | benperidol | -0.509 | 4 | -0.702 | 0.01647 | 0.0123 | 75 |
| 34 | mefloquine | 0.325 | 5 | 0.643 | 0.0165 | 0.1912 | 60 |
| 35 | F0447-0125 | 0.521 | 4 | 0.691 | 0.01898 | 0.0667 | 75 |
| 36 | furazolidone | 0.405 | 4 | 0.689 | 0.01963 | 0.0774 | 50 |
| 37 | vinpocetine | -0.223 | 4 | -0.684 | 0.02176 | 0.0318 | 50 |
| 38 | lactobionic acid | 0.473 | 4 | 0.677 | 0.02381 | 0.0074 | 75 |
| 40 | dosulepin | 0.424 | 4 | 0.676 | 0.02421 | 0.0564 | 75 |
| 41 | oxolamine | 0.357 | 4 | 0.675 | 0.02441 | 0.0989 | 50 |
| 42 | bisoprolol | -0.269 | 4 | -0.669 | 0.02715 | 0.0166 | 50 |
| 43 | simvastatin | -0.418 | 4 | -0.668 | 0.02737 | 0.0533 | 75 |
| 44 | 5155877 | 0.46 | 4 | 0.666 | 0.02813 | 0.1438 | 75 |
| 49 | alvespimycin | 0.352 | 12 | 0.397 | 0.03069 | 0.2759 | 50 |
| 50 | metyrapone | -0.48 | 4 | -0.658 | 0.03113 | 0.0529 | 75 |
| 51 | tobramycin | -0.313 | 4 | -0.655 | 0.03288 | 0.0955 | 50 |
| 52 | aminohippuric acid | -0.248 | 4 | -0.653 | 0.03378 | 0.0787 | 50 |
| 53 | zoxazolamine | -0.474 | 4 | -0.649 | 0.03593 | 0.101 | 75 |
| 54 | carteolol | 0.298 | 4 | 0.647 | 0.03692 | 0.0839 | 50 |
| 55 | nitrofural | 0.297 | 4 | 0.638 | 0.04178 | 0.0392 | 50 |
| 56 | glycocholic acid | 0.446 | 4 | 0.638 | 0.04221 | 0.0214 | 75 |

**Supplementary Table S4.** List of candidate molecules from the connectivity map queried by metabolic-related differentially expressed genes (DEGs) by MV4-11-XR vs MV4-11

| **Rank** | **Cmap molecules** | **Mean**  **Score** | **No of**  **instances** | **Enrichment** | **P value** | **Specificity** | **Percent non-null** |
| --- | --- | --- | --- | --- | --- | --- | --- |
| 1 | phenoxybenzamine | -0.778 | 4 | -0.97 | 0 | 0.0091 | 100 |
| 2 | sirolimus | 0.301 | 44 | 0.456 | 0 | 0.0843 | 52 |
| 5 | wortmannin | 0.266 | 18 | 0.485 | 0.00022 | 0.1484 | 61 |
| 6 | amikacin | 0.468 | 4 | 0.877 | 0.0003 | 0.0089 | 100 |
| 7 | 0317956-0000 | 0.208 | 8 | 0.637 | 0.00112 | 0 | 62 |
| 8 | parthenolide | -0.693 | 4 | -0.816 | 0.00209 | 0.0483 | 100 |
| 9 | pyrvinium | -0.523 | 6 | -0.691 | 0.00211 | 0.0928 | 83 |
| 11 | cloxacillin | 0.239 | 4 | 0.787 | 0.004 | 0.0068 | 50 |
| 12 | helveticoside | -0.296 | 6 | -0.665 | 0.00401 | 0.026 | 50 |
| 13 | nifuroxazide | -0.573 | 4 | -0.786 | 0.00426 | 0.0213 | 100 |
| 15 | norcyclobenzaprine | -0.437 | 4 | -0.777 | 0.00511 | 0.031 | 75 |
| 16 | thiostrepton | -0.39 | 4 | -0.769 | 0.00573 | 0.0463 | 75 |
| 19 | thioridazine | -0.286 | 20 | -0.363 | 0.00722 | 0.3226 | 50 |
| 20 | troglitazone | 0.258 | 16 | 0.4 | 0.00754 | 0.0179 | 56 |
| 21 | isoconazole | -0.434 | 5 | -0.668 | 0.00949 | 0.0082 | 80 |
| 22 | vorinostat | 0.164 | 12 | 0.448 | 0.01002 | 0.5729 | 58 |
| 23 | dihydrostreptomycin | -0.372 | 5 | -0.659 | 0.01107 | 0 | 80 |
| 25 | pizotifen | -0.543 | 4 | -0.726 | 0.01148 | 0.0172 | 75 |
| 27 | mestranol | -0.499 | 4 | -0.72 | 0.01243 | 0 | 75 |
| 28 | bepridil | -0.241 | 4 | -0.717 | 0.01287 | 0.0455 | 50 |
| 29 | kaempferol | -0.512 | 4 | -0.714 | 0.01353 | 0.025 | 75 |
| 30 | geldanamycin | -0.23 | 15 | -0.392 | 0.01362 | 0.2422 | 60 |
| 31 | gramine | -0.239 | 4 | -0.713 | 0.01369 | 0.0328 | 50 |
| 33 | eldeline | -0.299 | 6 | -0.595 | 0.01549 | 0.0349 | 50 |
| 34 | astemizole | -0.466 | 5 | -0.637 | 0.01604 | 0.1386 | 80 |
| 35 | mefloquine | -0.446 | 5 | -0.633 | 0.01704 | 0.1389 | 80 |
| 36 | tonzonium bromide | -0.445 | 4 | -0.697 | 0.01784 | 0.0776 | 75 |
| 37 | bendroflumethiazide | -0.325 | 6 | -0.585 | 0.01784 | 0.023 | 66 |
| 38 | withaferin A | -0.527 | 4 | -0.694 | 0.01846 | 0.1393 | 75 |
| 39 | dosulepin | -0.519 | 4 | -0.694 | 0.01852 | 0.0171 | 75 |
| 40 | mafenide | -0.416 | 5 | -0.626 | 0.019 | 0.0299 | 80 |
| 41 | ellipticine | 0.409 | 4 | 0.691 | 0.01902 | 0.1879 | 75 |
| 42 | prenylamine | -0.268 | 4 | -0.691 | 0.01959 | 0.0826 | 50 |
| 43 | amrinone | 0.29 | 4 | 0.682 | 0.0219 | 0.0221 | 50 |
| 44 | cicloheximide | 0.407 | 4 | 0.679 | 0.02308 | 0.1977 | 75 |
| 46 | Chicago Sky Blue 6B | 0.205 | 4 | 0.676 | 0.02401 | 0.0727 | 50 |
| 48 | butyl hydroxybenzoate | -0.4 | 5 | -0.611 | 0.02487 | 0.0272 | 60 |
| 49 | promazine | -0.248 | 6 | -0.564 | 0.0256 | 0.0423 | 50 |
| 50 | nitrofurantoin | -0.435 | 5 | -0.604 | 0.0273 | 0.0247 | 80 |
| 51 | clioquinol | -0.519 | 5 | -0.603 | 0.02766 | 0.0413 | 80 |
| 52 | vanoxerine | -0.413 | 4 | -0.665 | 0.02857 | 0.0417 | 75 |
| 53 | 5194442 | -0.49 | 4 | -0.663 | 0.02894 | 0.0573 | 75 |
| 54 | securinine | -0.463 | 4 | -0.659 | 0.03062 | 0.1646 | 75 |
| 56 | megestrol | -0.429 | 4 | -0.656 | 0.03223 | 0.0204 | 75 |

**Supplementary Table S5.** Antibodies for immunoblotting

| **Product** | **Vendor** | **Catalog no. #** | **Host** |
| --- | --- | --- | --- |
| Phospho-FLT3 (Tyr589/Tyr591) | Cell Signaling | #3464 | Rabbit |
| FLT3 | Santa Cruz | Sc-479 | Mouse |
| Phospho-STAT5 (Tyr694) | Genetex | GTX61079 | Rabbit |
| STAT5 | Genetex | GTX61098 | Rabbit |
| Phospho-AKT(Ser473) | Cell Signaling | #4058 | Rabbit |
| AKT | Cell Signaling | #9272 | Rabbit |
| Phospho-p44/42 ERK 1/2 (Thr202/Tyr204) | Cell Signaling | #4370 | Rabbit |
| p44/42 ERK1/2 | Cell Signaling | #4695 | Rabbit |
| p-CREB(Ser133) | Cell signaling | #9198 | Rabbit |
| CREB | Cell signaling | #9197 | Rabbit |
| GAPDH | Genetex | GTX627408 | Mouse |
| β-actin | Genetex | GTX109639 | Rabbit |
| α-tubulin | Genetex | GTX628802 | Mouse |

**Supplementary Table S6.** Primer sequences

Sequencing primers

| Gene | Primer sequence (5'→3') | Description |
| --- | --- | --- |
| cDNA |  |  |
| *FLT3* (E13-E22) | F – TCCCTTGGCACATCTTGTGA  R – GGAATGCCAGGGTAAGGAT | Annealing temp:  58℃ |
| DNA |  |  |
| *FLT3* (E19-I19) | F – CCGGGAAGATAATGCGATAG  R – TGGAACTGCAGACACACACC | Annealing temp:  58℃ |
| *FLT3* (I19-E20) | F – TGCCTTGAGCAACCAAGTAG  R – GCAGACTGCTGTGAGGGTTTT | Annealing temp:  58℃ |
| *FLT3* (E20-I20) | F – CCTTCCATCACCGGTACCTC  R – GCACCCAGCCAGGTATTGTA | Annealing temp:  58℃ |
| *FLT3* (I20-E21) | F – GTGCTGTGAAGGACTCGTCA  R – GGAATGCCAGGGTAAGGAT | Annealing temp:  58℃ |
| *FLT3* (I19-I20) | F – TGTTCCCCTTCAGATCTTCC  R – GCACCCAGCCAGGTATTGTA | Annealing temp:  58℃ |

*E: exon, I: intron

Primers for pyrosequencing

| Gene | Primer sequence (5'→3') | Description |
| --- | --- | --- |
| *FLT3* | F – CCTTCCATCACCGGTACCTC  R – Biotin – GCAGACTGCTGTGAGGGTTTT | Annealing temp:  58℃ |
| *FLT3* D835Y | F – GATATGTGACTTTGGATTGGC | Sequencing primer |

Primers for GeneScan

| Gene | Primer sequence (5'→3') | Description |
| --- | --- | --- |
| *FLT3* | F – CAAATCTCAGGGCTTCATGG  R – FAM-GGAATGCCAGGGTAAGGAT | Annealing temp:  58℃ |

RT-q-PCR primers

| Gene | Primer sequence (5'→3') | Description |
| --- | --- | --- |
| *18s rRNA* | F – GTAACCCGTTGAACCCCATT  R – CCATCCAATCGGTAGTAGCG |  |
| *HIF1A* | F – CATAAAGTCTGCAACATGGAAGGT  R – ATTTGATGGGTGAGGAATGGGTT |  |
| *GLUT1* | F – AGGTGATCGAGGAGTTCTAC  R – TCAAAGGACTTGCCCAGTTT |  |
| *GLUT5 (SLC2A5)* | F – TCTGTAACCGTGTCCATGTTTC  R – CATTAAGATCGCAGGCACGATA |  |
| *HK2* | F – CAAAGTGACAGTGGGTGTGG  R – TCAAAGGACTTGCCCAGTTT |  |
| *PKM2* | F – CCACTTGCAATTATTTGAGGAA  R – GTGAGCAGACCTGCCAGACT |  |
| *LDHA* | F – AGCCCGATTCCGTTACCT  R – CACCAGCAACATTCATTCCA |  |
| *PDK1* | F – ACCAGGACAGCCAATACAAG  R – CCTCGGTCACTCATCTTCAC |  |
| *PPARGC1B* | F – GGCAGGCCTCAGATCTAAAA  R – TCATGGGAGCCTTCTTGTCT |  |
| *NRF1* | F – CCATCTGGTGGCCTGAAG  R – GTAGTGCCTGGGTCCATGA |  |
| *TFAM* | F – GAACAACTACCCATATTTAAAGCTCA  R – GAATCAGGAAGTTCCCTCCA |  |

Primers for mitochondrial DNA quantification

| Gene | Primer sequence (5'→3') | Description |
| --- | --- | --- |
| *tRNA-Leu(UUR)* | F – CACCCAAGAACAGGGTTTGT  R – TGGCCATGGGTATGTTGTTA |  |
| *B2M* | F – TGCTGTCTCCATGTTTGATGTATCT  R – TCTCTGCTCCCCACCTCTAAGT |  |

**Supplementary methods**

**Quantification of mitochondria mass**

To assess the mitochondria mass, total DNA was first isolated from the indicated cell lines by the DNeasy kits (Qiagen, Venlo, Netherlands), and the ratio between nuclear DNA and mitochondrial DNA was determined by qPCR with indicated primers (Supplementary Table S6).

**Protein phosphorylation measurement**

To evaluate the changes of protein phosphorylation in various cells, the Proteome Profiler Human Phospho-Kinase Array Kit (ARY005B; R&S Systems, Minneapolis, NE, USA) was used as per the manufacturer’s instructions. Briefly, a total of 1x10^7^ cells were harvested and washed once with cold PBS, and then lysed with lysis buffer (containing 10μg/μL aprotinin, 10μg/μL leupeptin, and 2μg/μL pepstatin A). The protein concentrations of cell lysates were quantified for measurement. After blocking with array buffer 1 for 1h, the membrane was incubated with 1μg protein of cell lysate in array buffer 1 overnight at 4°C. Next, the membrane was incubated with diluted Detection Antibody Cocktail for 2h at room temperature, washed, and then incubated with diluted Streptavidin-HRP for another 30min and washed. Finally, the chemiluminescent reagent mixture was applied on the membrane for developing chemiluminescent signals detected by LAS 4000.

**Whole-transcriptome sequencing (RNA-seq) and Transcriptomic analysis (SNV, fusion, gene expression)**

RNA extraction was performed as previously reported [1], and the library preparation and high parallel sequencing were conducted by Genomics company (Taipei, Taiwan). After trimming, FASTQ reads were aligned to reference genome hg19 by Bowtie2 [2], and the GATK pipeline of RNAseq short variant discovery (SNPs + Indels) [3] was used to identify genetic variants following variant annotation by Annovar [4]. Fusioncatcher [5] was used to screen out the gene translocation events/gene fusion. The potential genetic variants or fusion candidates were further validated by Sanger sequencing.

To quantify differential gene expression, trimmed reads were aligned to the reference genome hg19 by Rsubread (Bioconductor) [6] and the gene expression was evaluated by featureCounts [7]. The differentially-expressed genes (DEGs) between resistant cells and their parental cells were identified by EBSeq (Bioconductor) [8], with a threshold of ≥ 2-fold (or ≤ 0.5-fold) and a *p*-value of ≤ 0.05. The transcriptome files can be found in the Gene Expression Omnibus (GEO) database (GSE234724). Next, the pathway enrichment analysis was performed by gene set enrichment analysis (GSEA) [9] using the Molecular Signatures Database v7.0 (MSigDB, Broad Institute, Cambridge, MA, USA) and Metascape [10]. Expression level of genes was validated by quantitative reverse transcription-PCR (q-RT-PCR) according to methods previously described [11]. The primers used are listed in Supplementary Table S6.

**Quantification of variant allele frequency by pyrosequencer and DNA fragment analyzer**

The interested fragments of DNA or cDNA samples were first amplified by AmpliTaq Gold® DNA Polymerase with Buffer II and MgCl_2_ (Thermo Fisher Scientific) with indicated primers (Supplementary Table S6). Pyrosequencing was then conducted by PyroMark Q24 Vacuum workstation (Qiagen, Hilden, Germany). For fragment analysis, capillary electrophoresis was performed; diluted PCR products were mixed with formaldehyde and fluorescent size standard, denatured by 95°C for 2min before loading to 3730xl DNA Analyzer (Applied Biosystems; Waltham, MA, USA), and finally analyzed by GeneScan™ (Applied Biosystems).

**Supplementary Reference**

1. Fu YH, Tseng CY, Lu JW, Lu WH, Lan PQ, Chen CY, et al. Deciphering the Role of Pyrvinium Pamoate in the Generation of Integrated Stress Response and Modulation of Mitochondrial Function in Myeloid Leukemia Cells through Transcriptome Analysis. Biomedicines. 2021;9(12).

2. Langmead B, Salzberg SL. Fast gapped-read alignment with Bowtie 2. Nat Methods. 2012;9(4):357-9.

3. Poplin R, Ruano-Rubio V, DePristo MA, Fennell TJ, Carneiro MO, Auwera GAVd, et al. Scaling accurate genetic variant discovery to tens of thousands of samples. bioRxiv. 2018:201178.

4. Wang K, Li M, Hakonarson H. ANNOVAR: functional annotation of genetic variants from high-throughput sequencing data. Nucleic Acids Res. 2010;38(16):e164.

5. Nicorici D, Şatalan M, Edgren H, Kangaspeska S, Murumägi A, Kallioniemi O, et al. FusionCatcher – a tool for finding somatic fusion genes in paired-end RNA-sequencing data. bioRxiv. 2014:011650.

6. Liao Y, Smyth GK, Shi W. The R package Rsubread is easier, faster, cheaper and better for alignment and quantification of RNA sequencing reads. Nucleic Acids Res. 2019;47(8):e47.

7. Liao Y, Smyth GK, Shi W. featureCounts: an efficient general purpose program for assigning sequence reads to genomic features. Bioinformatics. 2014;30(7):923-30.

8. Leng N, Dawson JA, Thomson JA, Ruotti V, Rissman AI, Smits BM, et al. EBSeq: an empirical Bayes hierarchical model for inference in RNA-seq experiments. Bioinformatics. 2013;29(8):1035-43.

9. Subramanian A, Tamayo P, Mootha VK, Mukherjee S, Ebert BL, Gillette MA, et al. Gene set enrichment analysis: a knowledge-based approach for interpreting genome-wide expression profiles. Proceedings of the National Academy of Sciences of the United States of America. 2005;102(43):15545-50.

10. Zhou Y, Zhou B, Pache L, Chang M, Khodabakhshi AH, Tanaseichuk O, et al. Metascape provides a biologist-oriented resource for the analysis of systems-level datasets. Nature communications. 2019;10(1):1523.

11. Su KW, Ou DL, Fu YH, Tien HF, Hou HA, Lin LI. Repurposing cabozantinib with therapeutic potential in KIT-driven t(8;21) acute myeloid leukaemias. Cancer Gene Ther. 2022;29(5):519-32.
