# Supplementary material for: Modulating metabolic signatures to mitigate cabozantinib resistance in FLT3-ITD acute myeloid leukemia cell models
Source: Cell Death Discov. 2026 Feb 17;12:98. doi: 10.1038/s41420-026-02957-8 (PMC12920922; doi:10.1038/s41420-026-02957-8)

Related to Fig. 1E (1)

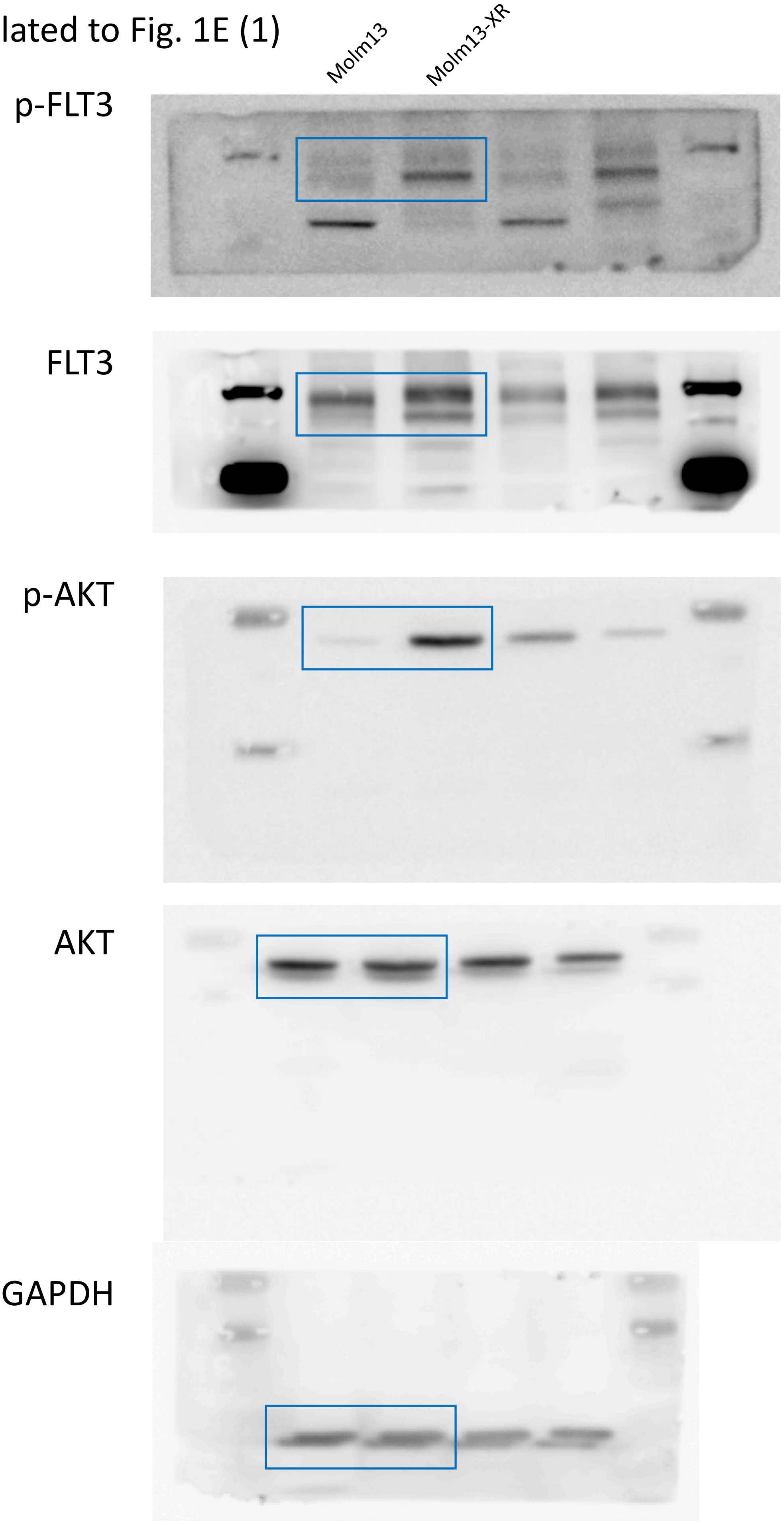

Related to Fig. 1E (2)

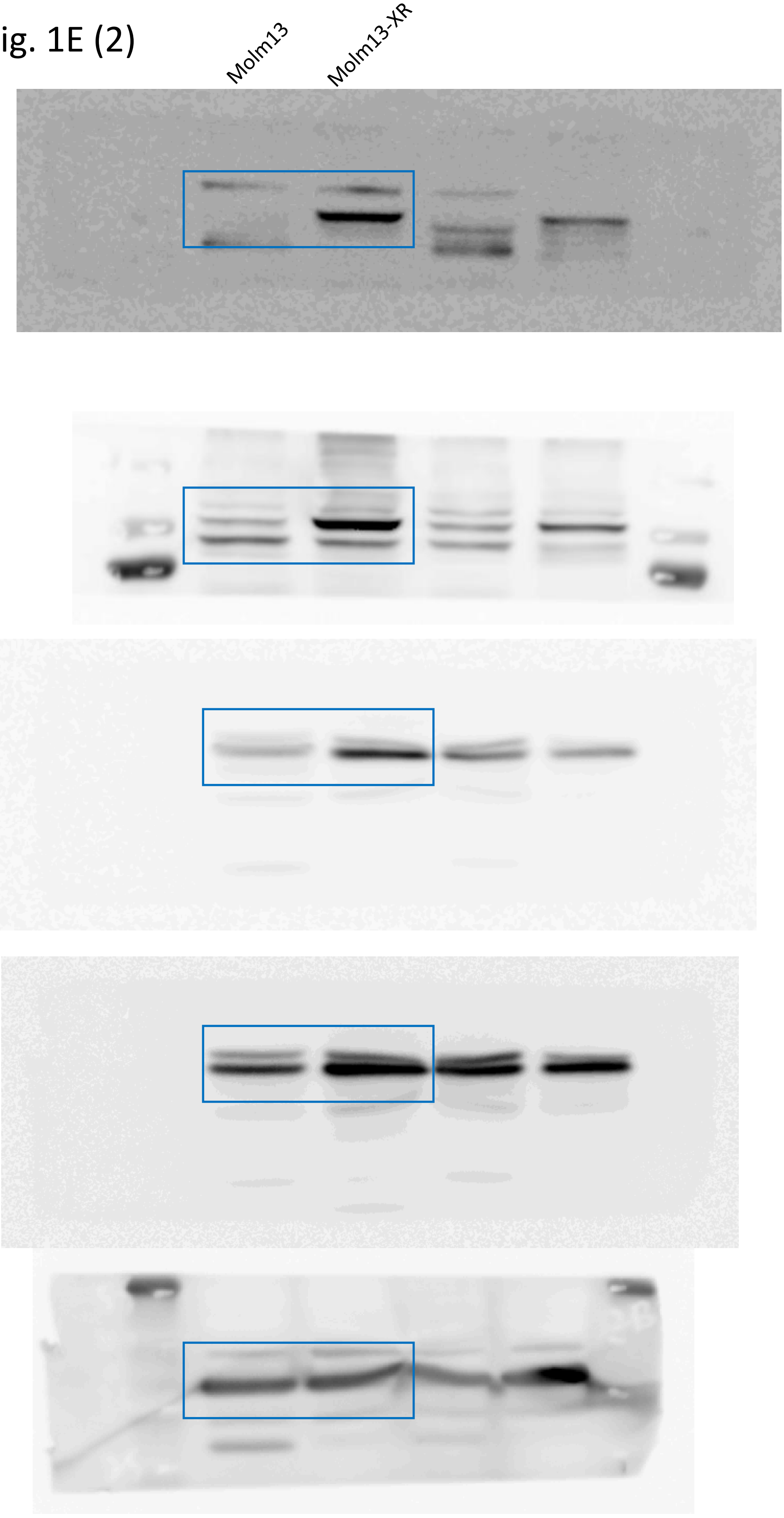

Related to Fig. 1E (3)

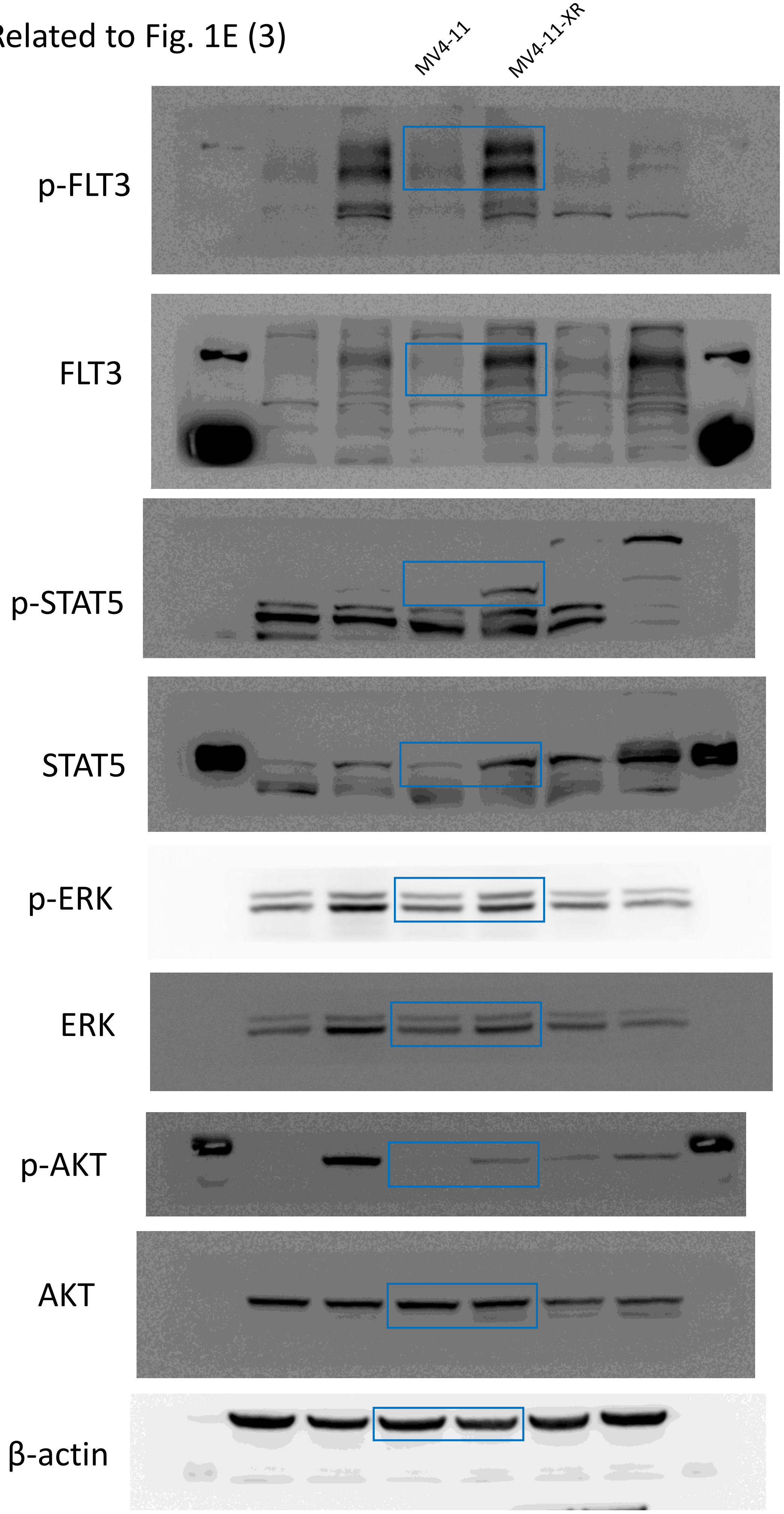

Related to Fig. 1F

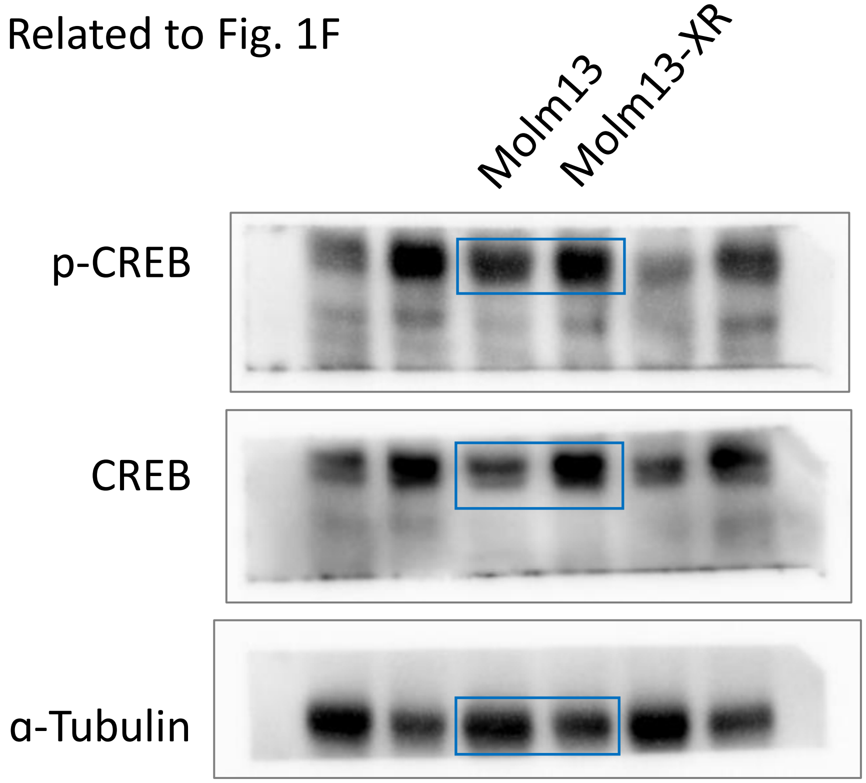

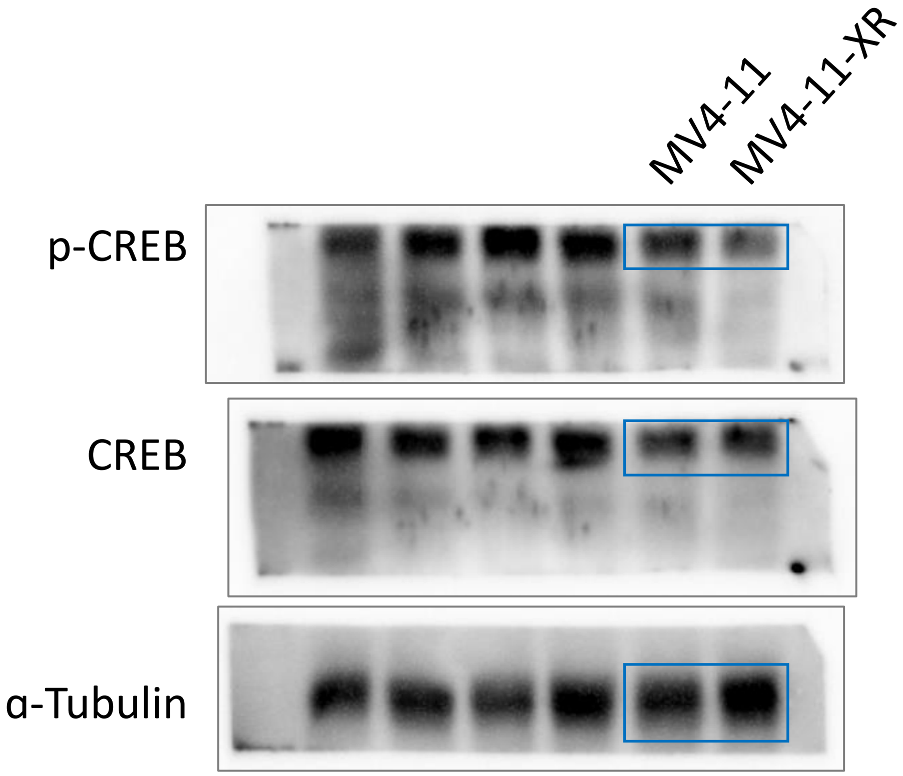

Supplement: Supplementary file 2 — Original Data Files [file 41420_2026_2957_MOESM2_ESM.pdf]
